# Spindle Dynamics during Meiotic Development of the Fungus Podospora anserina Requires the Endoplasmic Reticulum-Shaping Protein RTN1

**DOI:** 10.1128/mBio.01615-21

**Published:** 2021-10-05

**Authors:** Antonio de Jesús López-Fuentes, Karime Naid Nachón-Garduño, Fernando Suaste-Olmos, Ariadna Mendieta-Romero, Leonardo Peraza-Reyes

**Affiliations:** a Departamento de Bioquímica y Biología Estructural, Instituto de Fisiología Celular, Universidad Nacional Autónoma de México, Mexico City, Mexico; Karlsruhe Institute of Technology (KIT)

**Keywords:** endoplasmic reticulum (ER), meiosis, fungi, organelle dynamics, spindle, reticulon, sexual development, Spitzenkörper, organelle structure

## Abstract

The endoplasmic reticulum (ER) is an elaborate organelle composed of distinct structural and functional domains. ER structure and dynamics involve membrane-shaping proteins of the reticulon and Yop1/DP1 families, which promote membrane curvature and regulate ER shaping and remodeling. Here, we analyzed the function of the reticulon (RTN1) and Yop1 proteins (YOP1 and YOP2) of the model fungus Podospora anserina and their contribution to sexual development. We found that RTN1 and YOP2 localize to the peripheral ER and are enriched in the dynamic apical ER domains of the polarized growing hyphal region. We discovered that the formation of these domains is diminished in the absence of RTN1 or YOP2 and abolished in the absence of YOP1 and that hyphal growth is moderately reduced when *YOP1* is deleted in combination with *RTN1* and/or *YOP2*. In addition, we found that RTN1 associates with the Spitzenkörper. Moreover, RTN1 localization is regulated during meiotic development, where it accumulates at the apex of growing asci (meiocytes) during their differentiation and at their middle region during the subsequent meiotic progression. Furthermore, we discovered that loss of RTN1 affects ascospore (meiotic spore) formation, in a process that does not involve YOP1 or YOP2. Finally, we show that the defects in ascospore formation of *rtn1* mutants are associated with defective nuclear segregation and spindle dynamics throughout meiotic development. Our results show that sexual development in *P. anserina* involves a developmental remodeling of the ER that implicates the reticulon RTN1, which is required for meiotic nucleus segregation.

## INTRODUCTION

The endoplasmic reticulum (ER) is a multifaceted organelle that is composed of distinct structural and functional domains. The ER consists of a continuous membrane system composed of two major domains—the nuclear envelope (NE) and the peripheral ER—which enclose a common luminal space. The NE is formed by the outer and inner nuclear membranes, which fuse at the nuclear pores, while the peripheral ER consists of a complex network of tubules and cisternal sheets that extends from the NE throughout the cell (1). The ER constitutes the base of the cell endomembrane system and plays a fundamental role in the synthesis, processing, and transport of proteins and lipids (2). In addition, the ER has a central role in calcium homeostasis and signaling (3) and constitutes a hub that integrates complex signaling networks, which maintain cellular homeostasis and regulate cell fate (4–6). The ER participates in the biogenesis of multiple organelles (7) and establishes physical interactions with most membranous cell compartments, performing critical roles in the regulation of their activity and dynamics (1, 8, 9). Moreover, by conforming the NE, the ER is determinant in the regulation of nuclear assembly, dynamics, and remodeling (10–12).

ER structure and dynamics rely on a number of proteins that shape the organelle membranes, facilitate their homotypic fusion, and mediate their association with the cytoskeleton (1). Central to these processes are two conserved families of proteins—the reticulon and Yop1/DP1/REEP proteins—which form oligomers in the ER membrane that promote high membrane curvature (13, 14). Reticulons contain a conserved domain—the reticulon homology domain (RHD)—that is composed of two bipartite hydrophobic segments that form two hairpin-loops with a “W” topology in the ER membrane. Yop1 proteins possess a similar structural domain. These domains have been proposed to produce a hydrophobic wedge in the lipid bilayer promoting membrane bending (1, 15–17). In addition, these proteins possess a second domain required for membrane bending, which consists of a C-terminal amphipathic helix (18, 19). The reticulon and Yop1 proteins facilitate the formation of the peripheral ER tubules and stabilize the edges of ER sheets (14, 20–22). In addition, they have been proposed to stabilize the sites of fusion between the inner and outer membranes of the NE at the nuclear pores (23), and they participate in ER membrane constriction and fission (24).

The reticulon and Yop1/DP1 proteins have been implicated in diverse cellular roles, including the organization of the secretory pathway (25–27), the insertion of the nuclear pore complexes and spindle pole bodies (SPBs; the fungal nuclear membrane-embedded equivalents of centrosomes) in the NE (23, 28), and the regulation of ER segregation during cell division, which ensures inheritance of functional ER (29). Furthermore, the reticulons participate in the formation of the contact sites that the ER establishes with mitochondria (30, 31), peroxisomes (32), and the plasma membrane (33), as well as in the selective elimination of the ER via autophagy (34–37).

Fungi have provided significant insight into the molecular and cellular mechanisms conducting the developmental processes of sexual reproduction, such as karyogamy and meiosis (38–41). Research in fungi has also disclosed critical roles for the ER in these developmental processes. For example, in the yeast Saccharomyces cerevisiae karyogamy has long been known to depend on a number of proteins involved in ER protein import, folding, and quality control (42–45), whereas in filamentous ascomycetes the coordination of karyogamy with the meiotic program depends on a conserved ER SUN-domain protein (46). Nevertheless, there is still much to learn about the regulation of ER dynamics, as well as on the specific roles performed by the proteins that structure this organelle during sexual development. Here, we analyze the contribution of the reticulon and Yop1 proteins during sexual development of the model ascomycete Podospora anserina and show that the single reticulon protein of this fungus is required for meiotic nucleus segregation.

## RESULTS

### The *P. anserina* ER.

**FIG 1:**
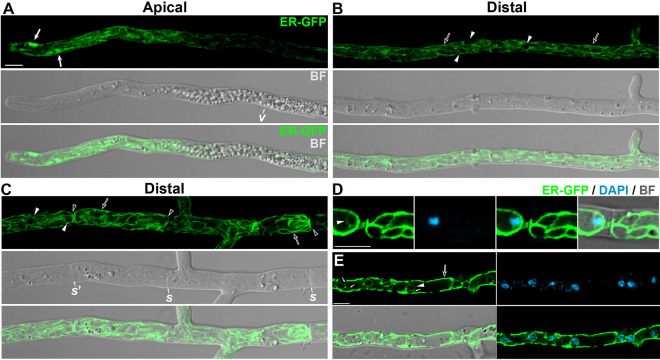
The Podospora anserina ER. Confocal microscopy analysis of ER-GFP-labeled ER in different hyphal regions of *P. anserina* young mycelial cultures (≈24 h old). The apical (A) and distal regions of leading hyphae extending approximately 100 μm (B) and 200 μm (C) behind the tip are shown. In panels D and E, the ER is compared to the localization of DAPI-stained nuclei in a leading hypha (D) and in a narrow hypha branching from distal hyphal regions (E). Arrows, apical ER subcompartments; arrowheads, nuclear envelope; open arrows, cortical ER; open arrowheads, cortical ER bordering septa; small arrows in panel E, ER strands connecting the cortical and nuclear ER; *v*, vacuolized hyphal region; *s*, septa; BF, bright field. Bar, 5 μm.

The *P. anserina* life cycle is shown in Fig. S1 in the supplemental material. In order to study the *P. anserina* ER, we analyzed the localization of an ectopically expressed ER-targeted green fluorescent protein (ER-GFP), which consisted of enhanced GFP (EGFP) flanked by the ER targeting and retention signals of the putative *P. anserina* ER chaperone BiP/Kar2 (47). We have shown that ER-GFP localizes to a large network of interconnected strands extending throughout the hypha (47), with a similar arrangement as that of the ER of other filamentous fungi (48–51). Moreover, we observed that ER-GFP exhibits a polarized distribution in hyphae, including an ER subdomain specifically located in the polarized growing apical region of hyphae (i.e., within the first ≈15 μm extending behind the hyphal tip). This ER subdomain consists of a number of dynamic and pleomorphic ER subcompartments (Fig. 1A), which are interconnected with the peripheral ER strands and which undergo fusion and division events accompanied by directional displacements along hyphae (47).

10.1128/mBio.01615-21.1 FIG S1 (A) Podospora anserina life cycle. *P. anserina* is a heterothallic ascomycete that reproduces exclusively sexually. Sexual reproduction takes place between genetically compatible strains differing in their mating type (denoted as *mat+* and *mat-*, respectively, and illustrated by nuclei [dots] with different shading) and involves the formation of multicellular fructifications known as perithecia. The perithecium arises from the fertilization of a female gametangium (ascogonium) by a male gamete (spermatium) and consists of a pyriform structure that encloses the fertile tissue (the hymenium) where karyogamy, meiosis, and ascospore (the meiosis-derived spore) formation occur. Ultimately, ascospores are expelled out of perithecia to germinate and produce a mycelium. Note that *P. anserina* produces binucleate (dikaryotic) and uninucleate (homokaryotic) ascospores (see below); for clarity, only the life cycle of homokaryotic strains issued from uninucleate ascospores is depicted. (B) *P. anserina* sexual development from the dikaryotic stage to ascospore formation (from left to right). The hymenium arises from the fertilized ascogonial cells, which differentiate specialized hook-shaped cells called croziers. In the crozier, the sorting and synchronized mitosis (lines connecting nuclei depict spindles) of a pair of opposite mating-type nuclei followed by the formation of septa in the site formerly occupied by the spindles produce a dikaryotic cell. This cell suffers karyogamy, enters meiosis, and differentiates into an ascus (the meiocyte). Asci then elongate from about 5 to more than 150 μm along meiotic prophase-I. Following meiosis, each nucleus divides mitotically to yield eight nuclei, which are subsequently packaged by pairs into four ascospores. Since the *mat*+/*mat*− alleles (idiomorphs) very frequently (≈98%) segregate at the second meiotic division, and because of the meiotic/postmeiotic spindle positioning, most dikaryotic ascospores produced possess opposite mating-type nuclei and yield, upon germination, a heterokaryotic self-fertile mycelium. This mating behavior is known as pseudohomothallism. In a low proportion of asci (≈2%), the change in the orientation of one of the postmeiotic mitosis spindles results in two distant nonsister nuclei, which are individually packaged into independent ascospores. This results in asci containing three binucleate ascospores and two smaller uninucleate ascospores. Ascospore differentiation and maturation are completed inside the original ascus. Download FIG S1, PDF file, 1.2 MB. Copyright © 2021 López-Fuentes et al. 2021 López-Fuentes et al. https://creativecommons.org/licenses/by/4.0/ This is an open-access article distributed under the terms of the Creative Commons Attribution 4.0 International license.

Additional ER subdomains also display a differential distribution along *P. anserina* hyphae. In growing leading hyphae, the apical ER region was followed by a segment with decreased ER-GFP fluorescence, located in the subapical hyphal region where large vacuoles accumulate (between 42.4 ± 9 and 84.4 ± 9 μm behind the hyphal tip, *n* = 15) (Fig. 1A), suggesting that the ER is partially excluded from this region by its high intracellular crowding. Behind this region, ER-GFP labeled a large network of ER strands throughout hyphae (Fig. 1B), which were more prominent toward the septate hyphal region (i.e., ≈250 μm behind the hyphal tip) (Fig. 1C). The ER-GFP-labeled ER network included a number of strands underlying the cell periphery, likely representing the plasma membrane-associated ER (Fig. 1B and C). In septate hyphal regions, these cortical ER strands also bordered the septa delimiting hyphal compartments (Fig. 1C). As previously described for the Aspergillus oryzae ER (49), septa were frequently flanked by parallel ER strands from adjacent compartments (Fig. 1C). However, we also observed continuity of the ER through the septal pores communicating between cell compartments (Movie S1). Consistent with localization to the NE, we found that ER-GFP also labeled the periphery of large spherical structures likely representing nuclei (Fig. 1B and C and Movie S1), as was corroborated by staining cells with 4′,6-diamidino-2-phenylindole (DAPI) (Fig. 1D and E). Finally, we found that the ER arrangement in narrow hyphae branching from distal regions of mycelia was characterized by prominent cortical ER strands interconnected with the NE by a number of short cytoplasmic strands (Fig. 1E).

10.1128/mBio.01615-21.5 MOVIE S1 ER flow across hyphal compartments. Live-cell imaging of the ER in the distal region of a growing hypha. (Top) Compared localizations of ER-GFP (green) and an FM4-64-labeled septum (red). (Bottom) ER-GFP/bright-field merge. Note the transfer across the septum of a large spherical structure likely representing a nucleus. Time scale is in seconds. Download Movie S1, MOV file, 0.6 MB. Copyright © 2021 López-Fuentes et al. 2021 López-Fuentes et al. https://creativecommons.org/licenses/by/4.0/ This is an open-access article distributed under the terms of the Creative Commons Attribution 4.0 International license.

### *P. anserina* possesses three proteins of the reticulon and Yop1 families.

Next, we searched for the genes potentially coding for proteins of the reticulon and Yop1 families in the *P. anserina* genome (52), and we identified one reticulon (*RTN1*) and two Yop1 (*YOP1* and *YOP2*) protein-encoding genes. The predicted RTN1 and YOP1 proteins possess characteristic RHD (Pfam02453) and TB2/DP1, HVA22 family (Pfam03134) domains, respectively. In contrast, YOP2 possesses an atypical N-terminal TB2/DP1, HVA22 domain, which bears only one transmembrane domain in its first hydrophobic segment (Fig. S2).

10.1128/mBio.01615-21.2 FIG S2 Sequences of the *P. anserina* proteins of the reticulon and Yop1 families. (A) Schematics of *P. anserina* RTN1, YOP1, and YOP2. Note that RTN1 and YOP1 possess characteristic RHD (Pfam02453) and TB2/DP1, HVA22 family (Pfam03134) domains, respectively, characterized by two large hydrophobic segments, each predictably composed of two tandem transmembrane domains. In contrast, YOP2 possesses an N-terminal TB2/DP1, HVA22 domain, in which only one transmembrane domain could be detected in the first of its two hydrophobic segments. (B) Alignment of RTN1 with representative proteins from fungi, humans, and plants. The reticulon homology domain (RHD) and the putative transmembrane-spanning regions (TM) of *P. anserina* RTN1 are overlined (in light and dark blue, respectively). The transmembrane segments of Saccharomyces cerevisiae (Y. Shibata, C. Voss, J. M. Rist, J. Hu, et al., J Biol Chem 283:18892–18904, 2008, https://doi.org/10.1074/jbc.M800986200), Homo sapiens (G. K. Voeltz, W. A. Prinz, Y. Shibata, J. M. Rist, and T. A. Rapoport, Cell 124:573–586, 2006, https://doi.org/10.1016/j.cell.2005.11.047; N. Zurek, L. Sparks, and G. Voeltz, Traffic 12:28–41, 2011, https://doi.org/10.1111/j.1600-0854.2010.01134.x), and Arabidopsis thaliana (E. Breeze, N. Dzimitrowicz, V. Kriechbaumer, R. Brooks, et al., Proc Natl Acad Sci U S A 113:10902–10907, 2016, https://doi.org/10.1073/pnas.1605434113; I. Sparkes, N. Tolley, I. Aller, J. Svozil, et al., Plant Cell 22:1333–1343, 2010, https://doi.org/10.1105/tpc.110.074385) proteins are underlined in green. GenBank accession numbers of the protein sequences utilized are as follows: Podospora anserina, CDP24655.1; Aspergillus nidulans, QDK59803.1; S. cerevisiae Rtn1p, AAS56905.1; H. sapiens reticulon-4 isoform C, NP_008939.1; *A. thaliana* Reticulon-like protein B13, NP_565555.1. (C) Alignment of *P. anserina* YOP1 family proteins with representative proteins from fungi, humans, and plants. The TB2_DP1_HVA22 family domain (red) and the putative transmembrane-spanning regions (dark blue) of *P. anserina* YOP1 and YOP2 are overlined. The transmembrane segments of S. cerevisiae Yop1p (J. P. Brady, J. K. Claridge, P. G. Smith, and J. R. Schnell, Proc Natl Acad Sci U S A 112:E639–E648, 2015, https://doi.org/10.1073/pnas.1415882112) are underlined (green). GenBank accession numbers of the protein sequences utilized are as follows: *P. anserina* (Pa) YOP1, CDP25565.1; YOP2, CDP28238.1; A. nidulans (An) AN2279.2 (Yop1), XP_659883.1; AN6059.4 (Yop2), CBF70269.1; S. cerevisiae (Sc) Yop1p, NP_015353.1; Yarrowia lipolytica (Yl) YALI0_B19668g (Yop1), XP_501105.2; YALI0_E05841g (Yop2), XP_503603.1; H. sapiens (Hs) DP1, AAA60136.1,; REEP1 isoform 2, NP_075063.1; *A. thaliana* (At) HVA22 homologue D, NP_567713.1; HVA22-like protein i, NP_568606.1. Sequence alignments were performed using MUSCLE (R. C. Edgar, Nucleic Acids Res 32:1792–1797, 2004, https://doi.org/10.1093/nar/gkh340). Identical amino acids are shaded in black (shade threshold 60%). The proteins were analyzed for conserved domains on the Pfam database (pfam.xfam.org), and the putative transmembrane-spanning regions were predicted using TOPCONS (K. D. Tsirigos, C. Peters, N. Shu, L. Kall, and A. Elofsson, Nucleic Acids Res 43:W401–W407, 2015, https://doi.org/10.1093/nar/gkv485), with similar results using Philius (S. M. Reynolds, L. Kall, M. E. Riffle, J. A. Bilmes, and W. S. Noble, PLoS Comput Biol 4:e1000213, 2008, https://doi.org/10.1371/journal.pcbi.1000213) (not shown). Download FIG S2, PDF file, 0.2 MB. Copyright © 2021 López-Fuentes et al. 2021 López-Fuentes et al. https://creativecommons.org/licenses/by/4.0/ This is an open-access article distributed under the terms of the Creative Commons Attribution 4.0 International license.

### YOP2 localizes to distinct ER domains and is enriched in the apical ER subcompartments.

**FIG 2:**
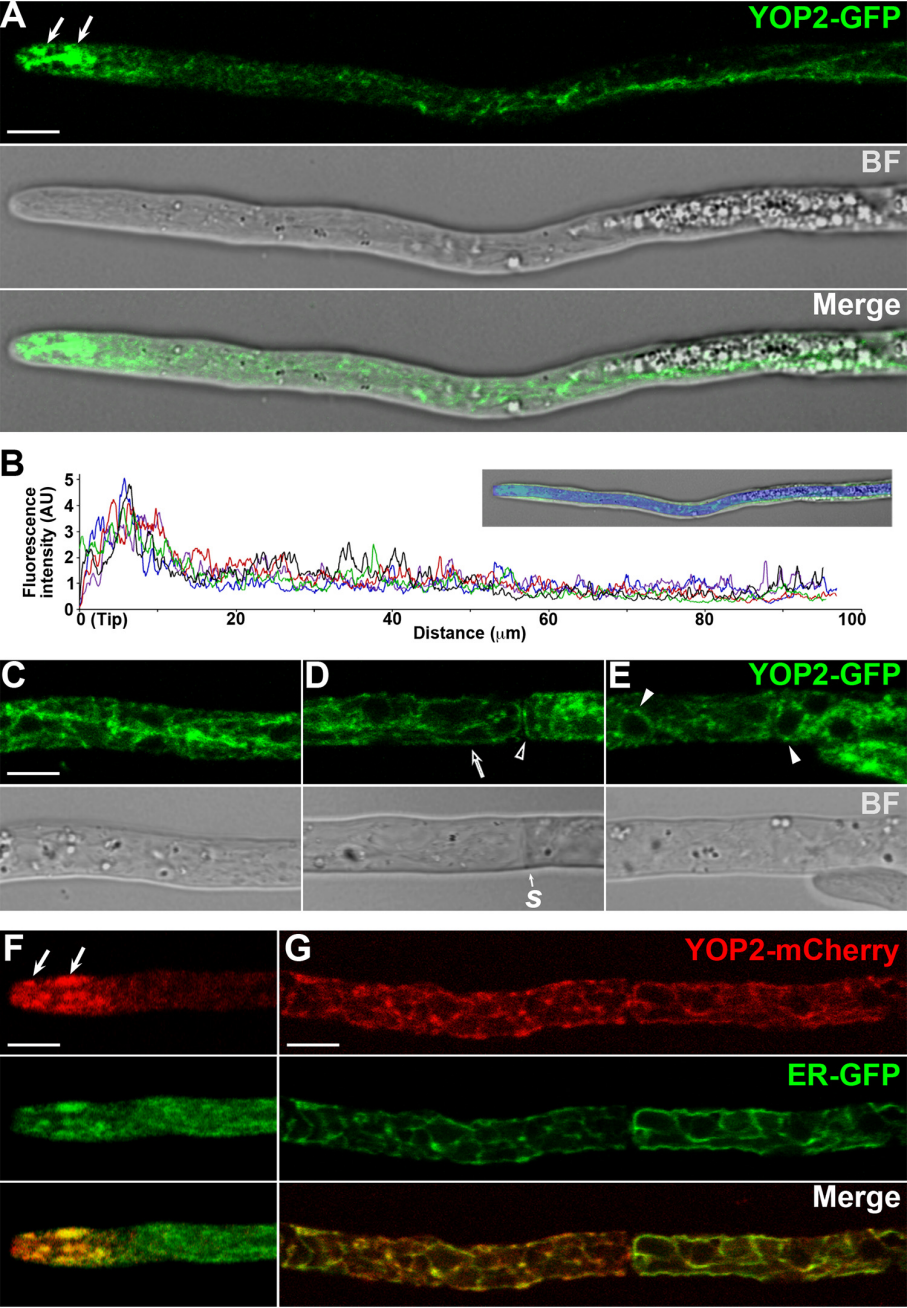
Localization of YOP2 in vegetative hyphae. (A) Localization of YOP2-GFP in the apical segment of growing leading hypha of young mycelial cultures. (B) Line scan graph of the fluorescence intensity profile of YOP2-GFP along growing leading hyphae; each line represents the line scan of an independent hypha. The upper panel shows an example of the line scan (blue) used to determine the intensity profile. (C to E) YOP2-GFP localization in distal regions of leading hypha. Note that the imaging parameters were optimized for YOP2-GFP detection in these regions and differ from panel A. (F and G) Localization of YOP2-mCherry and ER-GFP in apical (F) and distal (G) regions of hyphae. Arrows point to apical ER subcompartments, arrowheads to the nuclear envelope, open arrows to cortical ER, and open arrowheads to cortical ER bordering septa (*s*). BF, bright field; AU, arbitrary units. Bar, 5 μm.

A search for YOP1 orthologues in fungal genomes identified S. cerevisiae Yop1p as the orthologue of *P. anserina* YOP1. In contrast, we did not identify orthologues of *YOP2* in most Saccharomycotina fungi (with the exception of Yarrowia lipolytica). Nonetheless, *YOP2* orthologues were present throughout filamentous ascomycetes (Pezizomycotina), as well as in most basidiomycete fungi (Agaricomycotina and Pucciniomycotina), and in selected sequenced genomes available for early-diverging fungi (Blastocladiomycota, Chytridiomycota, and Mucormycota). These observations indicate a wide distribution for *YOP2* in fungi; nonetheless, no YOP2 protein has been previously characterized. Therefore, we first sought to define whether *P. anserina* YOP2 actually localizes to the ER. We generated strains expressing an mCherry C-terminally tagged version of YOP2 by tagging the *YOP2* gene at its chromosomal location. YOP2-mCherry exhibited dim fluorescence; therefore, by the same strategy we also generated strains expressing GFP-tagged YOP2. We did not observe growth or developmental defects in the strains expressing the YOP2 tagged proteins. YOP2-GFP exhibited a polarized distribution in hyphae, with higher fluorescence intensity in the apical region (Fig. 2A and B and Movie S2). In this region, YOP2-GFP predominantly localized to a number of pleomorphic patches (Fig. 2A, arrows) similar to the apical ER subcompartments labeled by ER-GFP. In subapical cells, YOP2-GFP localized to a network of strands, which were similar to those labeled by ER-GFP but more discontinuous and limited in extension (Fig. 2A and C). In addition, YOP2-GFP localized to the cortical ER (Fig. 2D) and faintly labeled the NE with a patchy distribution (Fig. 2E). Despite its weak fluorescence, YOP2-mCherry colocalized with ER-GFP at both the apical patches (Fig. 2F) and subapical strands (Fig. 2G) in double-label experiments. These observations indicate that YOP2 resides in different domains of the ER, with a prominent localization in the apical ER subcompartments.

10.1128/mBio.01615-21.6 MOVIE S2 Localization of YOP2-GFP in a growing hypha. Live-cell imaging of a growing leading hypha expressing YOP2-GFP (top). (Bottom) Corresponding bright field. Time scale is in minutes:seconds. Download Movie S2, MOV file, 9.8 MB. Copyright © 2021 López-Fuentes et al. 2021 López-Fuentes et al. https://creativecommons.org/licenses/by/4.0/ This is an open-access article distributed under the terms of the Creative Commons Attribution 4.0 International license.

### RTN1 localizes to the peripheral ER and is enriched in the apical ER subcompartments.

**FIG 3:**
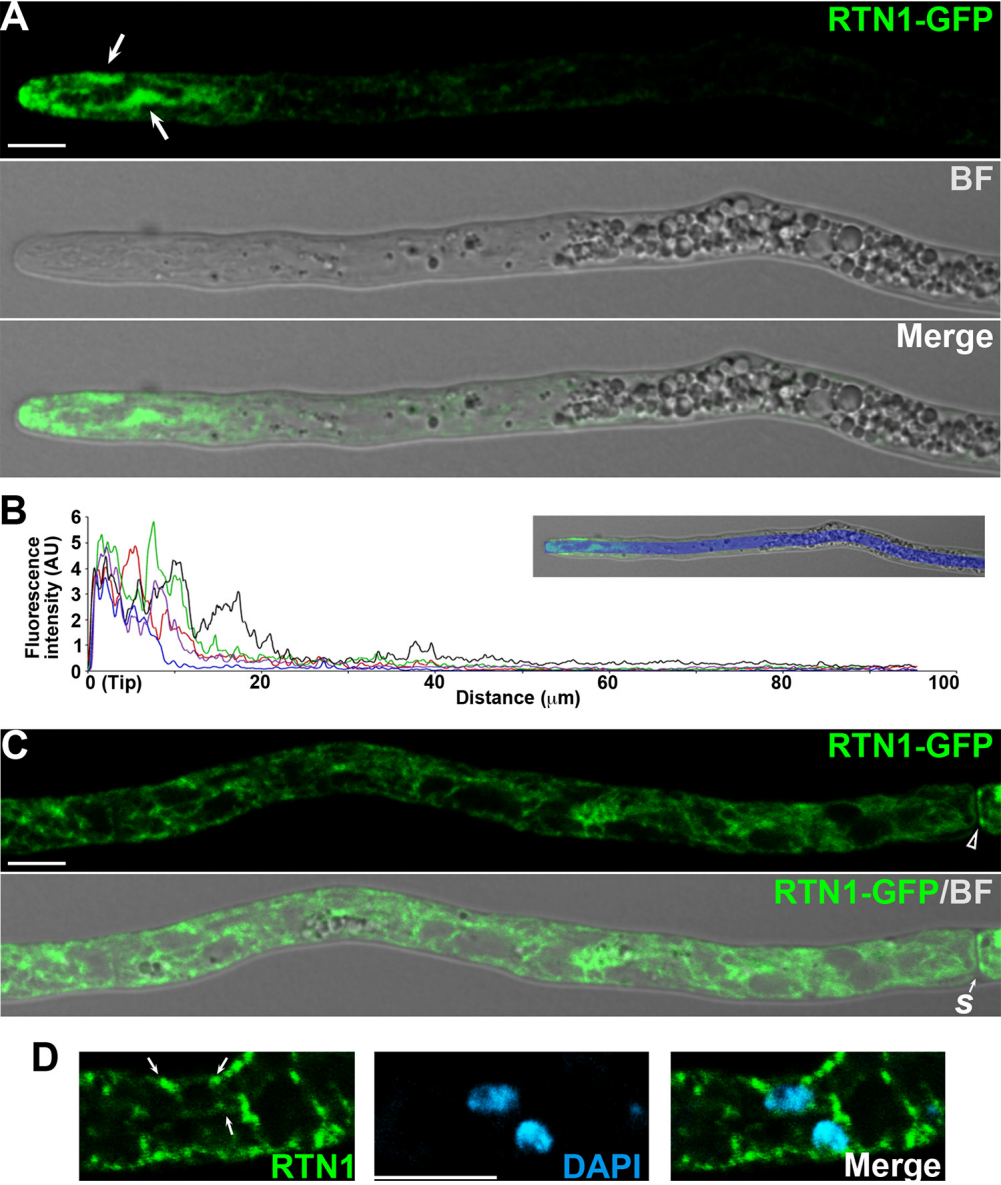
Localization of RTN1 in vegetative hyphae. (A) RTN1-GFP localization in the apical region of growing leading hyphae of young mycelial cultures. Arrows point to apical ER subcompartments. (B) Line scan graph of the fluorescence intensity profile of RTN1-GFP along growing leading hyphae; each line represents the analysis of an independent hypha. The upper panel shows an example of the line scan (blue) used to determine the intensity profile. (C) RTN1-GFP localization in the distal region of a leading hypha. The open arrowhead shows the cortical ER bordering septa (*s*). Note that to enhance RTN1-GFP detection, the imaging parameters differ from panel A. (D) Localization of RTN1-GFP in relation to nuclei (DAPI). Small arrows point to ER strands neighboring nuclei. BF, bright field; AU, arbitrary units. Bar, 5 μm.

Next, we studied the localization of RTN1 by generating strains expressing a GFP C-terminally tagged version of RTN1 by tagging the *RTN1* gene at its native locus. These strains exhibited the wild-type phenotype, including normal ascospore formation in homozygous crosses (Fig. S3; note that ascospore formation is affected in *rtn1* mutants; see below), indicating that RTN1-GFP is a functional protein. We found that, similarly to YOP2, RTN1-GFP exhibited a polarized localization in hyphae with increased fluorescence in the apical region (Fig. 3A and B and Movie S3), where it predominantly localized to the apical ER subcompartments (arrows). In subapical hyphal regions, RTN1-GFP less strongly labeled peripheral ER strands (Fig. 3C), including strands of the cortical ER (arrowhead). RTN1-GFP also labeled short strands in the vicinity of nuclei (Fig. 3D), but no distinctive NE labeling by RTN1-GFP was observed.

10.1128/mBio.01615-21.3 FIG S3 Genetic complementation of Δ*rtn1* strains and analysis of the strains expressing RTN1 tagged with GFP. Analysis of ascospore formation in homozygous sexual crosses of WT (A) and Δ*rtn1* (B) strains and in heterozygous crosses of Δ*rtn1* to Δ*rtn1* strains complemented with a wild-type *RTN1*^+^ allele (Δ*rtn1*+*RTN1*^+^; three independent transformants are shown) (C to E). Bar, 20 μm. (F) Quantitation of the abnormal asci produced in WT, Δ*rtn1* and *RTN1*::*GFP* homozygous crosses, and in heterozygous crosses of Δ*rtn1* to Δ*rtn1* strains complemented with a wild-type *RTN1*^+^ allele (Δ*rtn1*+*RTN1*^+^; three independent transformants were analyzed) or with a *GFP*::*RTN1*^+^ allele (*n* = 300 from three independent experiments). *, *P* < 0.0001 by one-way ANOVA with Tukey’s *post hoc* test. (G) Localization of GFP-RTN1 expressed in an *RTN1*^+^ growing leading hypha. (H) Compared apical localizations of GFP-RTN1 and the FM4-64-stained Spitzenkörper. Arrow points to the Spitzenkörper. BF, bright field. Bar, 5 μm. Download FIG S3, PDF file, 2.4 MB. Copyright © 2021 López-Fuentes et al. 2021 López-Fuentes et al. https://creativecommons.org/licenses/by/4.0/ This is an open-access article distributed under the terms of the Creative Commons Attribution 4.0 International license.

10.1128/mBio.01615-21.7 MOVIE S3 Localization of RTN1-GFP in a growing hypha. Live-cell imaging of a growing leading hypha expressing RTN1-GFP (top). (Middle) Bright field; (bottom) merge. Time scale is in minutes:seconds. Download Movie S3, MOV file, 6.0 MB. Copyright © 2021 López-Fuentes et al. 2021 López-Fuentes et al. https://creativecommons.org/licenses/by/4.0/ This is an open-access article distributed under the terms of the Creative Commons Attribution 4.0 International license.

### RTN1-GFP associates with the Spitzenkörper.

**FIG 4:**
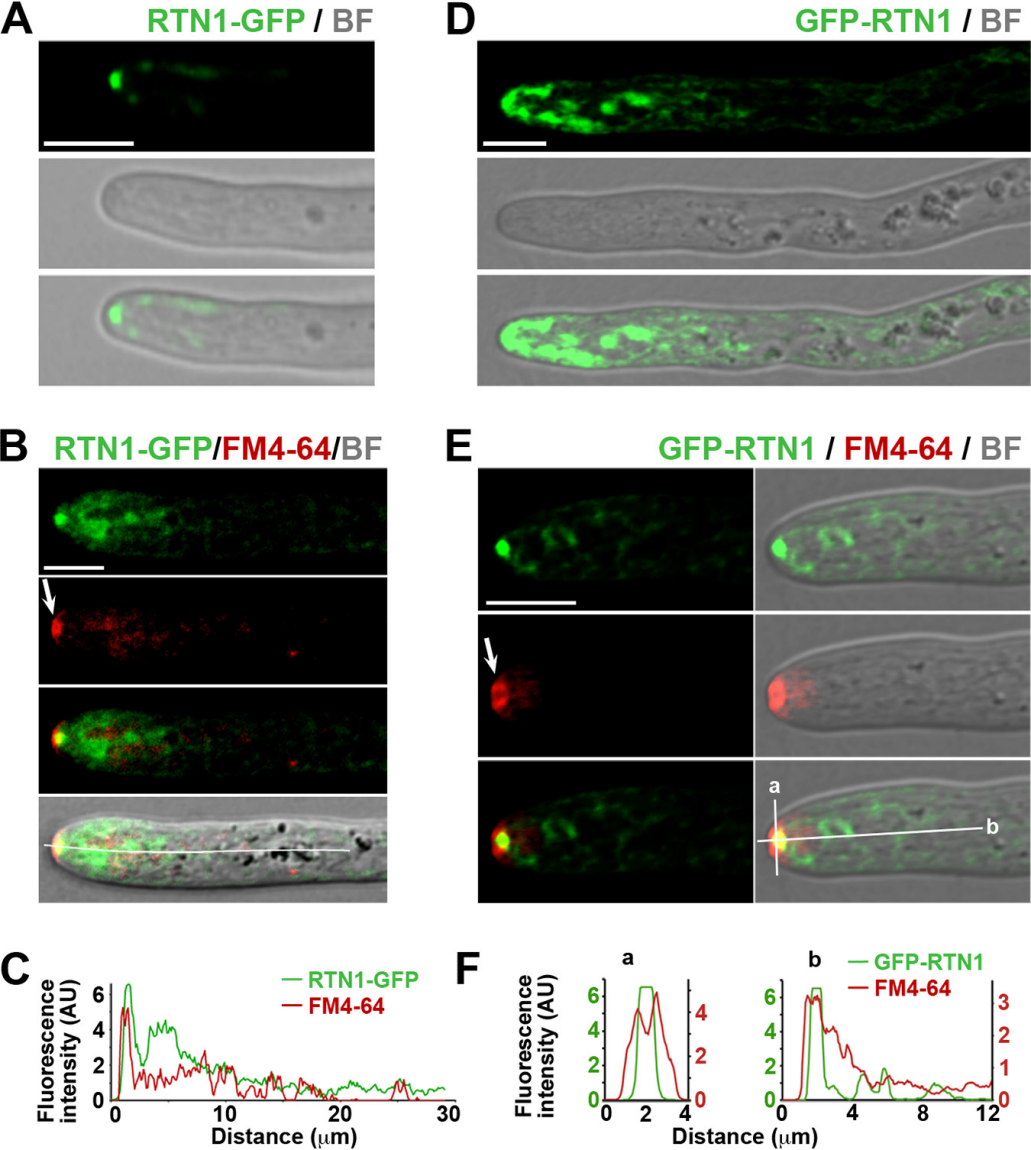
RTN1 localizes to the Spitzenkörper. Apical localization of RTN1-GFP (A) and compared localizations of RTN1-GFP and the FM4-64-stained Spitzenkörper (B) in growing leading hyphae of young mycelial cultures. (C) Fluorescence intensity profile of RTN1-GFP and FM4-64 along the hypha depicted in panel B (white line). (D and E) Localization of GFP-RTN1 expressed in a *Δrtn1* growing leading hypha (D) and compared apical localizations of GFP-RTN1 and the FM4-64-stained Spitzenkörper (E). (F) Fluorescence intensity profile of GFP-RTN1 and FM4-64 along the lines depicted in panel E, lines a and b, respectively. Note that the imaging parameters of panel D were enhanced to detect GFP-RTN1 at the apical subcompartments. Arrows point to the Spitzenkörper. BF, bright field; AU, arbitrary units. Bar, 5 μm.

In addition to the peripheral ER, we found that an apical punctum located at the hyphal tip was strongly stained by RTN1-GFP (Fig. 3A and Fig. 4A). This structure displayed poleward movement, which correlated with the extension of the hyphal tip (Movie S3), and its dynamics was reminiscent of the Spitzenkörper, a dynamic apical body conducting hyphal polar growth and morphogenesis. The Spitzenkörper is proposed to act as a vesicle supply center, which concentrates vesicles that transport cell wall-synthesizing enzymes for their subsequent delivery to the apical membrane, facilitating localized cell surface expansion at the hyphal tip (53, 54). We analyzed the Spitzenkörper by staining growing cells with FM4-64—a lipophilic fluorescent dye that labels this structure (54)—and found that RTN1-GFP was associated with the Spitzenkörper (Fig. 4B and C). The presence of the Spitzenkörper in a living hypha is associated with its elongation, and its position at the apex correlates with the hyphal growth direction. Actually, we observed that the appearance and disappearance events, and the changes in position of the FM4-64-stained Spitzenkörper, which were associated with hyphal growth and directionality, were mirrored by RTN1-GFP tip localization (Movie S4), corroborating its dynamic association with the Spitzenkörper. To rule out that the Spitzenkörper localization of RTN1 was artificially produced by the C-terminal tagging, we also analyzed the localization of a GFP N-terminally tagged version of RTN1. For this, we ectopically expressed a *GFP*::*RTN1* gene in cells deleted for the *RTN1* gene (Δ*rtn1*, see below). GFP-RTN1 was able to complement the Δ*rtn1* ascospore formation defects (Fig. S3), corroborating that this version of RTN1 is also functional. GFP-RTN1 showed the same characteristic distribution of RTN1-GFP, including its prominent localization at the apical ER subcompartments and at the Spitzenkörper (Fig. 4D to F and Movie S5). In addition, we observed that GFP-RTN1 displayed this same localization pattern when *GFP*::*RTN1* was expressed in the wild-type (*RTN1*^+^) genetic context (Fig. S3 and Movie S5). Of note, we observed that, in contrast to the annular appearance of the FM4-64-stained Spitzenkörper, RTN1 localized to a discrete punctum of this structure (Fig. 4B, C, E, and F and Fig. S3), denoting that it localizes to the Spitzenkörper core.

10.1128/mBio.01615-21.8 MOVIE S4 Compared localizations of RTN1-GFP and the Spitzenkörper in a growing hypha. Live-cell imaging of a growing leading hypha expressing RTN1-GFP and stained with FM4-64. Panels (from top to bottom): (i) RTN1-GFP, (ii) FM4-64, (iii) RTN1-GFP/FM4-64 merge, (iv) FM4-64/bright-field merge. Note that the hyphal growth pauses at the time that the Spitzenkörper disappears and then resumes in a new direction in the region where the Spitzenkörper reappears. Time scale is in minutes:seconds. Download Movie S4, MOV file, 6.2 MB. Copyright © 2021 López-Fuentes et al. 2021 López-Fuentes et al. https://creativecommons.org/licenses/by/4.0/ This is an open-access article distributed under the terms of the Creative Commons Attribution 4.0 International license.

10.1128/mBio.01615-21.9 MOVIE S5 Localization of GFP-RTN1 in hyphae. Live-cell imaging of growing leading hyphae expressing GFP-RTN1 in the Δ*rtn1* (first segment) or *RTN1*^+^ (second segment) genetic contexts. (Top panel) RTN1-GFP; (middle) bright field; (bottom) merge. Note that the imaging parameters of the segments differ, as they were enhanced to detect GFP-RTN1 at the apical subcompartments in the second segment. In the first segment, note the changes in position of the GFP-RTN1-labeled apical puncta (Spitzenkörper), which are followed by changes in the hyphal growth direction. Bar, 2 μm. In the second segment, note the dynamics of the apical ER subcompartments, which undergo directed displacements and fusion and fission events. Bar, 5 μm. Time scale: minutes:seconds. Download Movie S5, MOV file, 18.5 MB. Copyright © 2021 López-Fuentes et al. 2021 López-Fuentes et al. https://creativecommons.org/licenses/by/4.0/ This is an open-access article distributed under the terms of the Creative Commons Attribution 4.0 International license.

### The mycelial growth is moderately reduced in the absence of RTN1, YOP1, and YOP2.

**FIG 5:**
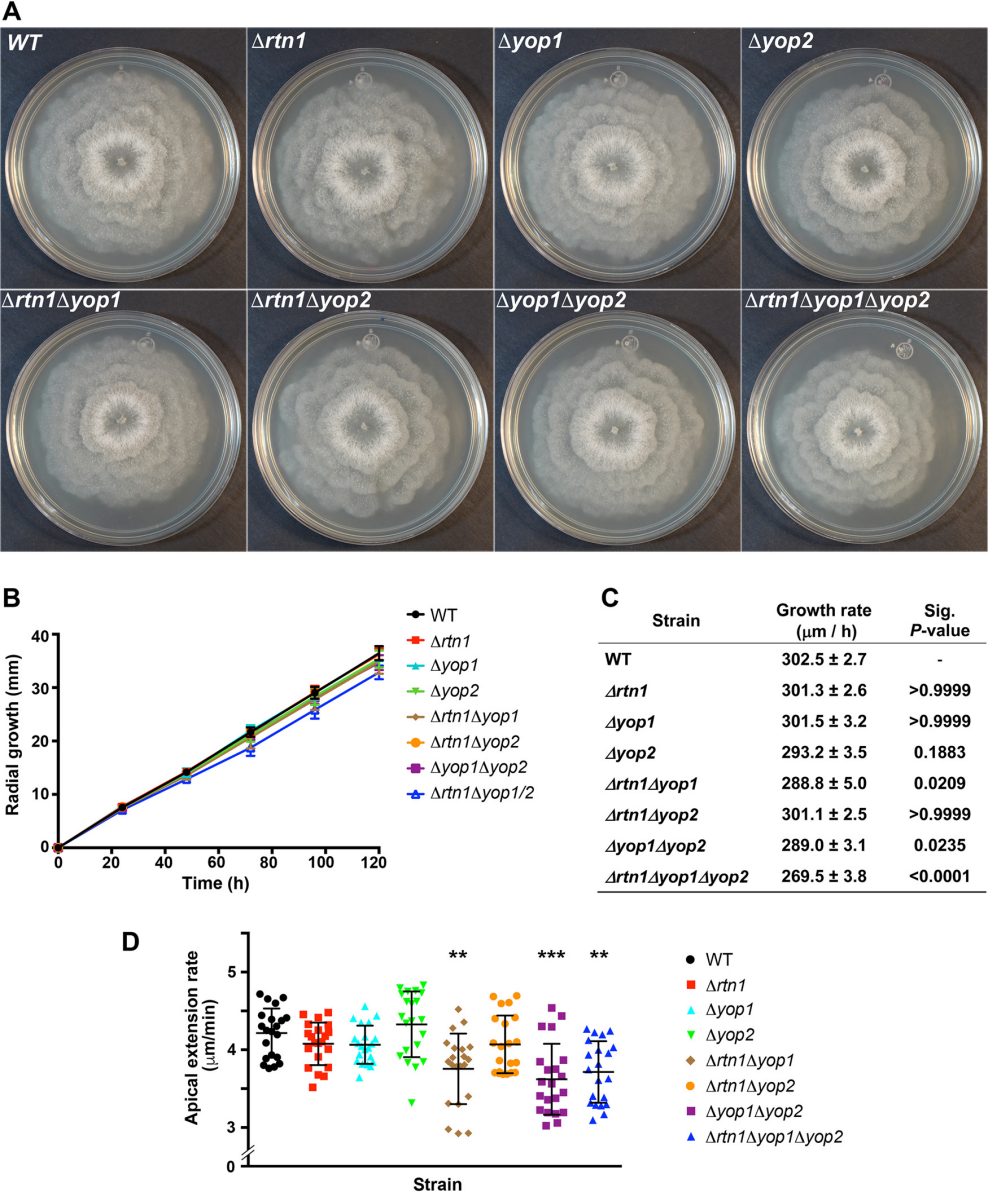
Growth phenotypes of *P. anserina* strains deficient for ER-shaping proteins. Mycelial growth of wild-type (WT) and deletion mutant strains deficient for the ER-shaping proteins RTN1, YOP1, and/or YOP2 on dextrin-containing medium. (A) Colonial growth at 120 h. (B) Growth curves. Values are mean ± SD from three independent experiments per strain, each with triplicates. (C) Growth rates of the strains analyzed in panel B; values are mean ± SEM. Statistically significant differences were evaluated by two-way ANOVA using a Tukey *post hoc* test; the adjusted *P* values of the mutant strains against the wild type are presented (Sig.). (D) Quantification of the apical extension rate of individual growing leading hyphae of the indicated strains. Scatterplots show the mean ± SD; *n* = 21 hyphae (from 3 independent experiments; 7 hyphae/experiment). **, *P* < 0.01; ***, *P* < 0.0001 relative to the WT by two-way ANOVA with Tukey’s *post hoc* test.

Next, we studied the function of YOP1, YOP2, and RTN1 by generating strains deleted for their corresponding genes. First, we observed that the *P. anserina* mycelial growth on standard dextrin-based medium was not significantly affected by the elimination of either of these genes (Fig. 5A to C). Then, we generated double and triple mutant strains deleted for these genes in all different combinations, and we observed a slight mycelial growth rate reduction in the double mutants involving Δ*yop1* (Δ*yop1* Δ*yop2* and Δ*rtn1* Δ*yop1* [Fig. 5]), as well a moderate reduction (i.e., ≈10%) in the *Δrtn1 Δyop1 Δyop2* triple mutant, compared to the wild type (Fig. 5A to C). In addition, we evaluated the effect of *RTN1*, *YOP1*, and *YOP2* deletion on the growth of individual hyphae. We analyzed the apical extension rate of growing leading hyphae by live-cell imaging and found that, similar to mycelia, it was not significantly affected by the single elimination of either RTN1, YOP1, or YOP2 (Fig. 5D). Nonetheless, we observed a moderate reduction in the apical extension rate of Δ*yop1* Δ*yop2* (average 3.62 ± 0.5 μm/min), Δ*rtn1* Δ*yop1* (average 3.76 ± 0.5 μm/min), and *Δrtn1 Δyop1 Δyop2* (average 3.71 ± 0.4 μm/min) mutant hyphae, compared to the wild type (average 4.21 ± 0.3 μm/min) (Fig. 5D). These observations suggest that the *P. anserina* reticulon and Yop1 proteins perform redundant functions required for optimal vegetative growth.

### ER structure depends to different extents on RTN1, YOP1, and YOP2.

**FIG 6:**
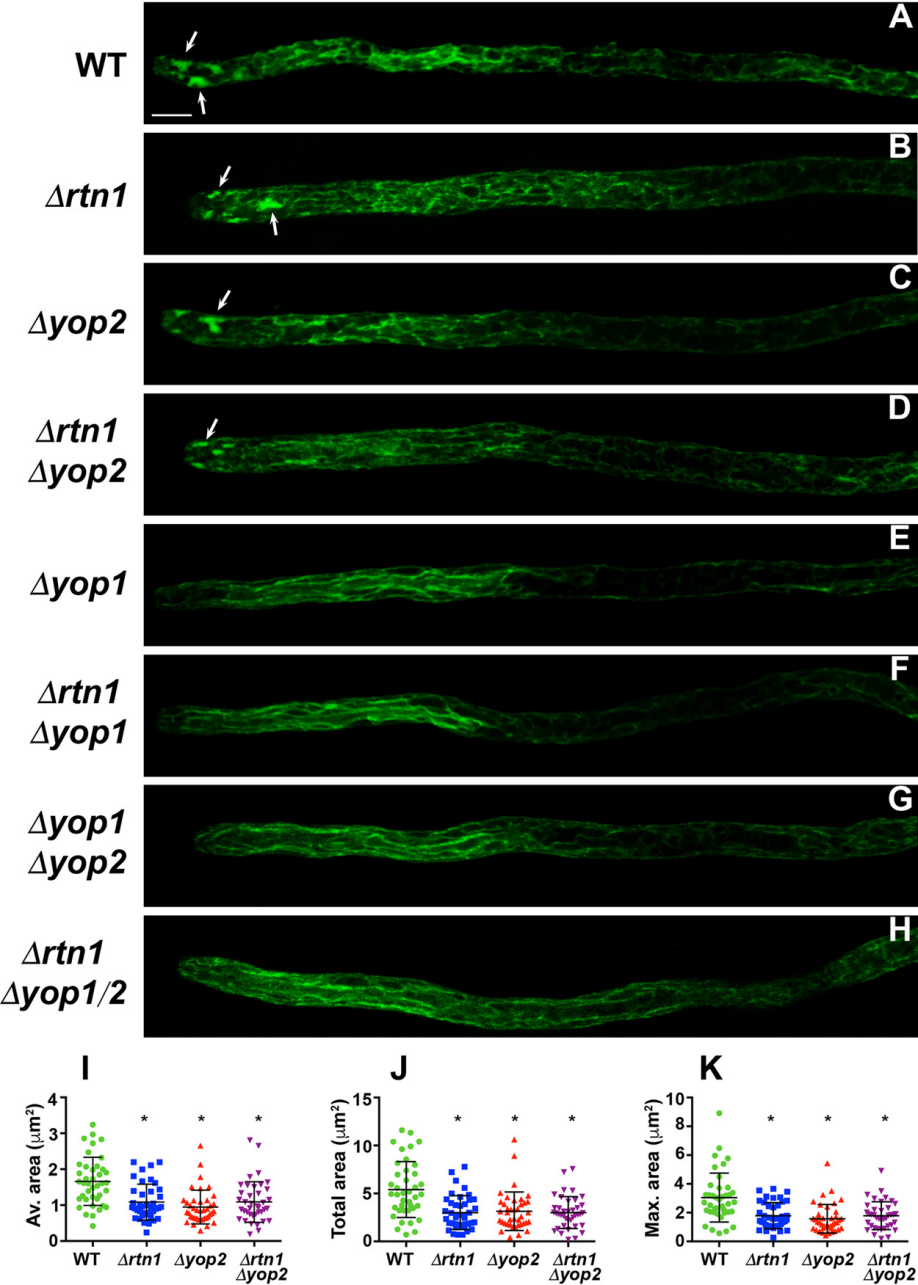
YOP1, YOP2, and RTN1 are required for ER shaping. (A to H) Analysis of ER-GFP-labeled ER in the apical region of growing leading hyphae of young mycelial cultures of strains of the indicated genotypes (WT, wild type). Arrows point to apical ER subcompartments. Bar, 5 μm. (I to K) Quantification of the average area of the apical subcompartments per hypha (I), of the total area occupied by all apical subcompartments in a hypha (J), and of the maximal area attained by an apical subcompartment per hypha (K) of WT, *Δrtn1*, *Δyop2*, and *Δrtn1 Δyop2* strains. Scatterplots show the mean ± SD; *n* = 40 hyphae (from 4 independent experiments; 10 hyphae/experiment). *, *P* < 0.0001 relative to the WT by two-way ANOVA with Tukey’s *post hoc* test.

We assessed the participation of YOP1, YOP2, and RTN1 in ER structuring by analyzing the localization of ER-GFP in the different deletion strains (Fig. 6A to H). We discovered that elimination of either RTN1 (Fig. 6B) or YOP2 (Fig. 6C) resulted in a decrease in the size of the apical ER subcompartments, relative to the wild type (Fig. 6A), as revealed by determining the average (Fig. 6I), the total (Fig. 6J), or the maximal (Fig. 6K) area occupied by the apical ER subcompartments in hyphae of the corresponding deletion mutants. We did not observe additive defects or suppression of this phenotypic trait upon simultaneously eliminating RTN1 and YOP2 (Fig. 6D and I to K). Interestingly, we found that the elimination of YOP1 by itself generated a more severe defect in the formation of the apical ER subcompartments, which were virtually undetectable in the *Δyop1* mutant (Fig. 6E). This defect was accompanied by a rearrangement of the peripheral ER of the subapical hyphal region, which was characterized by ER strands predominantly displaying a longitudinal arrangement and that appeared to be less branched (Fig. 6E). We found that *Δyop1* was epistatic on *Δrtn1* (Fig. 6F) and *Δyop2* (Fig. 6G) for this phenotype and that the triple *Δrtn1 Δyop1 Δyop2* mutant (Fig. 6H) displayed the same phenotype as the single *Δyop1* mutant. These observations indicate that the formation of the apical ER depends to a different extent on the activity of YOP1, YOP2, and RTN1, where YOP1 exerts a major contribution.

### The elimination of RTN1 affects sexual development.

Then, we analyzed the effect of *YOP1*, *YOP2*, and *RTN1* deletion on sexual development. *P. anserina* is a heterothallic ascomycete that reproduces exclusively sexually. Sexual development takes place inside multicellular fructifications (perithecia), which enclose the sexual tissue (the hymenium) where karyogamy, meiosis, and ascospore (the meiosis-derived spore) formation occur. This process involves the formation of a specialized ascogenous hypha—the crozier—where opposite mating-type nuclei are compartmentalized in a dikaryotic cell. This cell undergoes karyogamy, enters meiosis, and differentiates into an ascus (the meiocyte). The ascus elongates along meiotic prophase-I, and after ending meiosis, a mitotic division yields eight haploid nuclei, which are enclosed by pairs into four ascospores. Although formally heterothallic, *P. anserina* usually produces dikaryotic ascospores, which possess opposite mating-type nuclei and upon gemination yield a heterokaryotic mycelium that is able to self-fertilize. This reproductive strategy is referred to as pseudohomothallism. Ascospore differentiation and maturation are completed inside the original ascus (Fig. S1) (55, 56).

**FIG 7:**
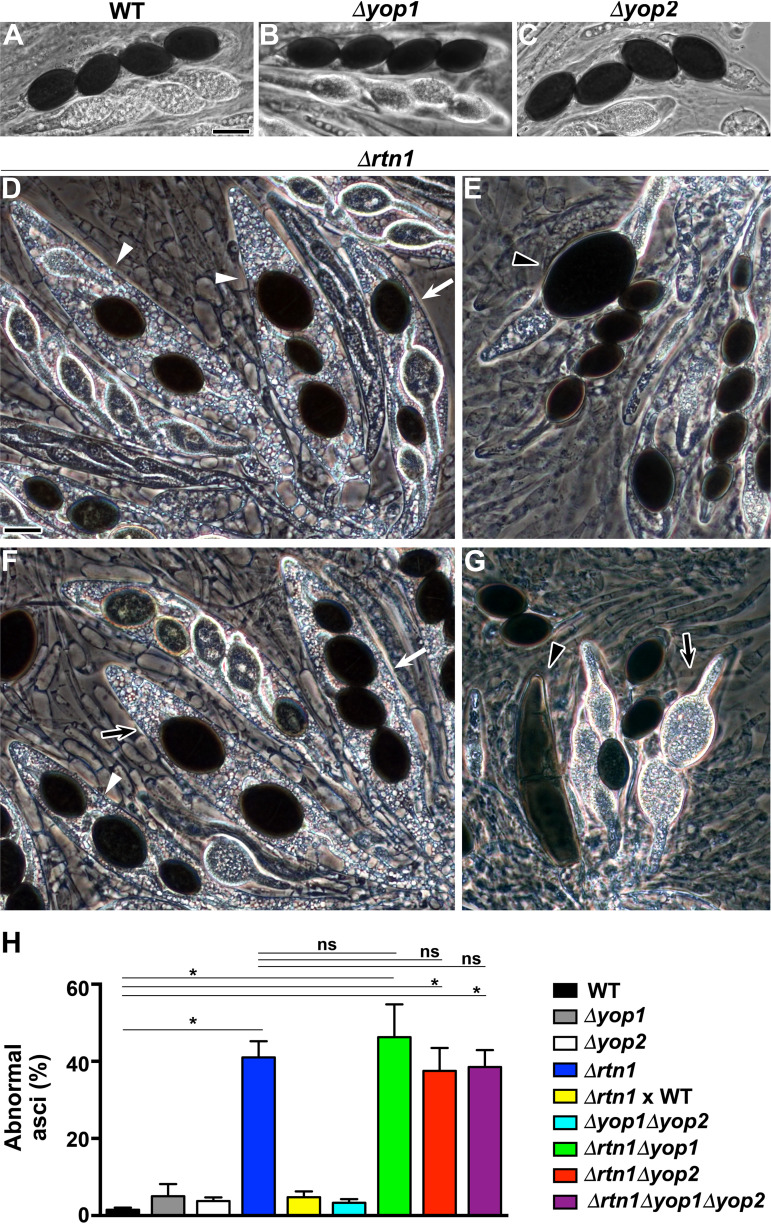
*RTN1* deletion affects ascospore formation. Asci issued from WT (A), *Δyop1* (B), *Δyop2* (C), and *Δrtn1* (D to G) homozygous crosses. Note that in contrast to the WT, *Δyop1*, and *Δyop2* asci, which contain four ascospores of even size, *Δrtn1* asci produce ascospores of uneven size (white arrows), in irregular numbers (black arrows; note an earlier developmental stage in panel G), and irregular in both size and number (white arrowheads). Eventually, *Δrtn1* asci containing a single giant ascospore, as well as a large banana-like ascospore (black arrowheads in panels E and G, respectively), were produced. Bar, 20 μm. (H) Quantitation of abnormal asci in the indicated sexual crosses. *Δrtn1* × WT corresponds to a heterozygous mutant × WT cross, and the remaining crosses correspond to homozygous crosses of the indicated genotypes (*n* = 400 asci from four independent experiments). *, *P* < 0.0001 by one-way ANOVA with Tukey’s *post hoc* test; ns, not statistically significant.

First, we analyzed sexual development in sexual crosses homozygous for *Δyop1*, *Δyop2*, or *Δrtn1*. We did not observe defects in the formation of perithecia in either genetic context, indicating that YOP1, YOP2, and RTN1 are not required for fertilization or perithecium formation. We also observed that perithecia issued from these crosses were able to produce ascospores (Fig. 7A to G), implying that YOP1, YOP2, and RTN1 are not strictly required for karyogamy, meiosis, or ascospore formation. Nonetheless, we observed that ascospores produced in *Δrtn1* homozygous crosses were abnormal (Fig. 7D to G). We found that, in contrast to the wild type, which primarily produces asci containing four ascospores of even size (Fig. 7A), *Δrtn1* perithecia frequently contained asci with ascospores of uneven sizes and in irregular numbers (Fig. 7D to G). Eventually, *Δrtn1* asci containing a single gigantic ascospore were produced (Fig. 7E), as well as asci producing a single large banana-shaped ascospore (Fig. 7G). These asci were observed at a very low frequency in the *Δrtn1* strain (i.e., in 0.6% of asci, *n* = 1,200 from 3 independent experiments); however, they were never observed in the wild type (*n* > 1,200). Overall, we found that around 40% (41% ± 4.2%) of asci issued from *Δrtn1* homozygous crosses were abnormal, compared to less than 2% (1.5% ± 5.8%) in the wild type (Fig. 7H). These defects were not observed in heterozygous crosses of *Δrtn1* to the wild type (Fig. 7H), indicating a recessive phenotype. This phenotype was also not observed in *Δyop1*, *Δyop2*, or *Δyop1 Δyop2* homozygous crosses (Fig. 7B, C, and H), and it was not exacerbated or suppressed upon deleting *YOP1* and/or *YOP2* in the *Δrtn1* context (Fig. 7H). Finally, we showed that the ectopic introduction of a wild-type allele of *RTN1* into the *Δrtn1* genetic background restored ascospore formation (Fig. S3), corroborating that this phenotype was caused by *RTN1* deletion.

### Ascospore germination is affected in the absence of RTN1.

Next, we inspected whether ascospore germination was affected by RTN1 loss. We found that 86% of *Δrtn1* ascospores (*n* = 102) were able to geminate when they were issued from morphologically normal *Δrtn1* asci (i.e., asci containing 4 ascospores of even size), compared to 98% in the wild type (*n* = 102). We also found that the *Δrtn1* germination rate was reduced to 55% when ascospores were issued from abnormal *Δrtn1* asci (*n* = 102 ascospores). In these asci, we found no correlation between the ascospore size and its germination capacity (i.e., the germination rates for ascospores of regular and abnormal size were 44% and 56%, respectively). These findings show that RTN1 is required for ascospore germination, in correlation with its influence in ascus development.

### RTN1 localization is regulated during meiotic development.

**FIG 8:**
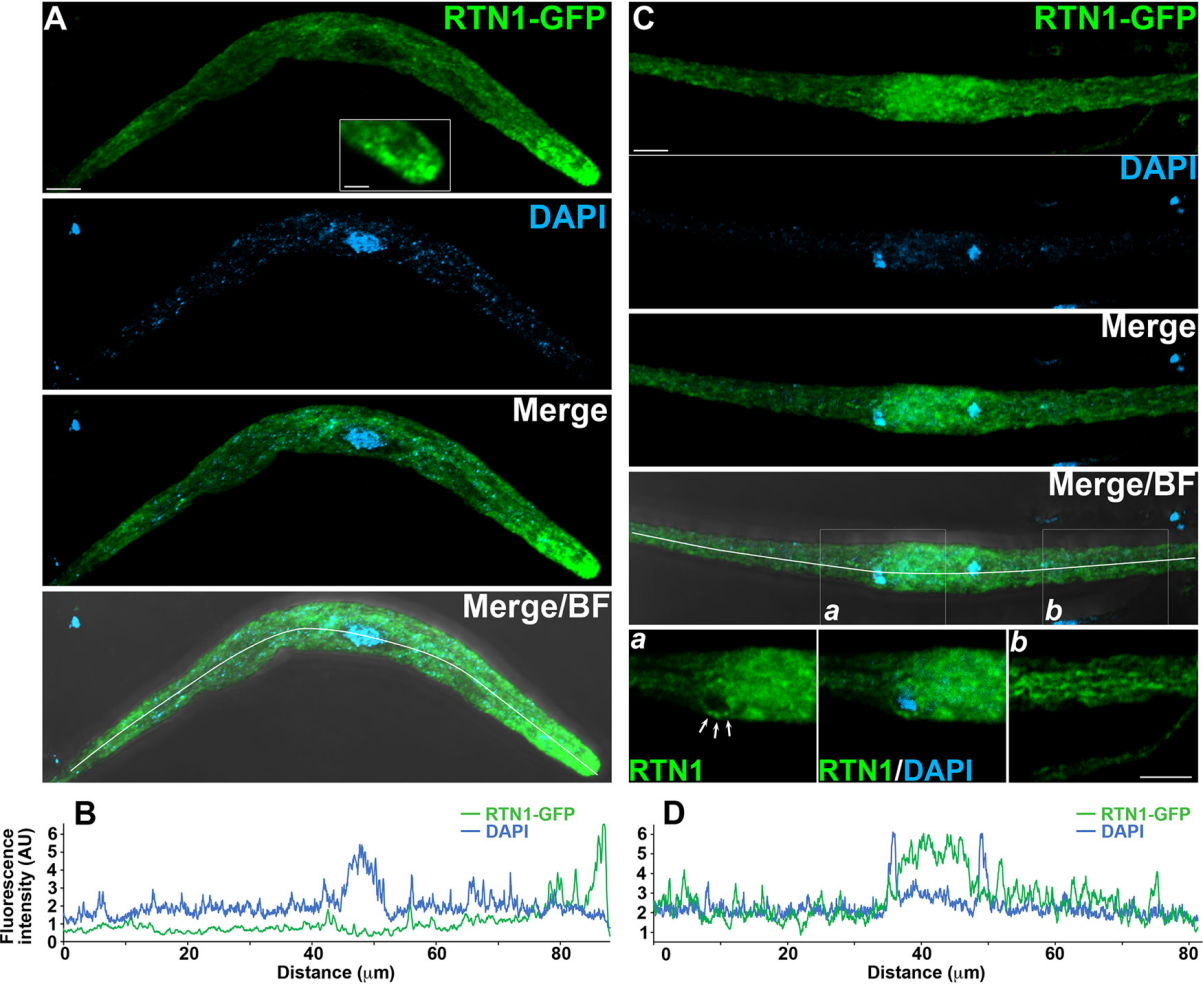
RTN1 localization is regulated during meiotic development. (A) RTN1-GFP localization in a late meiotic prophase-I ascus. The inset shows an enlarged single-plane micrograph of the ascus tip. (B) Line scan graph of the fluorescence intensity for RTN1-GFP (green) and DAPI (blue) channels along the ascus in panel A (white line). (C) RTN1-GFP localization in an ascus after the first meiotic division. Insets *a* and *b* display enlarged single-plane images of the corresponding boxed areas. In inset *a*, the localization of RTN1-GFP is compared to that of a nucleus (DAPI), and arrows point to RTN1-GFP foci at the nuclear periphery; inset *b* shows RTN1-GFP localization near the cell cortex. (D) Line scan graph of the fluorescence intensity for RTN1-GFP (green) and DAPI (blue) channels along the ascus in panel C (white line). Note the increase in RTN1-GFP fluorescence in the ascus middle region, which is bordered by the segregating nuclei. Except for the insets, images show maximum-intensity projections of z series through the entire cells. DNA was stained with DAPI. BF, bright field. Bars, 5 μm. Inset bar in panel A, 2 μm.

Then, we analyzed the localization of RTN1-GFP during ascus development. Similar to vegetative hyphae, we found that RTN1-GFP displayed a polarized distribution early during ascus development, at the stages where asci elongate along meiotic prophase-I (Fig. 8A and B). In these asci, RTN1-GFP displayed increased fluorescence toward the apex, where it localized to a number of apical patches (Fig. 8A, inset). This suggests that RTN1 also associates with a specialized ER subdomain in the polarized growing regions of this specialized hyphal cell type. Following meiotic prophase-I, when the ascus reached its final length, the intensity of RTN1-GFP fluorescence at the apical region decreased concomitantly with an increase in ascus middle region. RTN1-GFP prominently localized to the ascus middle region during meiotic progression, in the vicinity of the region where nuclei segregate (shown for an ascus after meiosis-I in Fig. 8C and D). Consistent with peripheral-ER localization, RTN1-GFP localized to a network of strands in these cells (Fig. 8C, inset *b*). However, we found that RTN1-GFP also localized to a number of small puncta bordering nuclei (Fig. 8C, inset *a*). These observations show that the localization of RTN1 is regulated during meiocyte development.

### Loss of RTN1 affects nucleus segregation during meiotic development.

**FIG 9:**
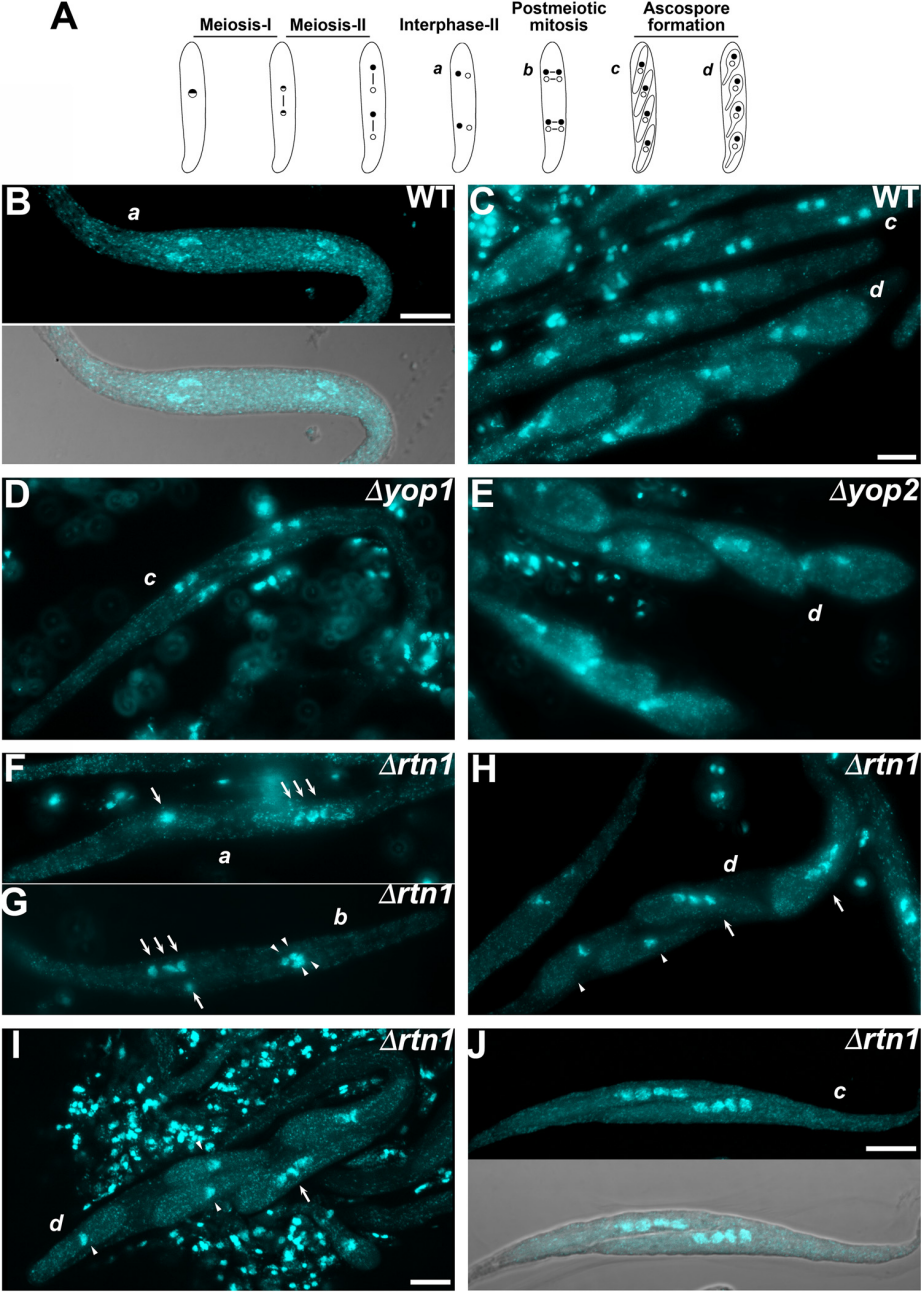
*RTN1* deletion affects the segregation of nuclei during meiotic development. (A) *P. anserina* meiotic development from late prophase-I to ascospore formation (from left to right). Dots represent nuclei, and the lines connecting dots represent spindles (see the text for details). Small lettering indicates successive developmental stages shown in the figure. (B to J) Nucleus distribution during meiotic development of WT (B and C), *Δyop1* (D), *Δyop2* (E), and *Δrtn1* (F to J) homozygous crosses. Wild-type meiosis results in four evenly distributed nuclei, which associate as pairs at the following interphase (B) to then divide across the ascus. This produces four pairs of nuclei, which are packaged into four ascospores (C; three asci at progressive developmental stages, from top to bottom, are seen, each containing four binucleate ascospores). *Δyop1* (D) and *Δyop2* (E) asci exhibited normal meiotic nucleus distribution. *Δrtn1* asci displayed alterations in the disposition of nuclei following meiosis (F; note the three clustered nuclei distant from a single isolated nucleus [arrows]; compare to panel B), at postmeiotic mitosis (G; note the three clustered nuclei next to a single nucleus [arrows], compared to two pairs of nuclei [arrowheads] at the opposite side of the ascus), and in ascospores (H to J; compare to panel C). Note that the abnormal meiotic nucleus disposition of panel F could yield an ascus with the uneven ascospore nuclear distribution of panel H. Also note the two tetranucleate ascospores in panel J, which upon growth and maturation could develop into the two large ascospores shown in Fig. 7G and F (black arrows), respectively. DNA was stained with DAPI. Lower panels in panels B and J show DAPI/bright-field merge images. Bars, 5 μm.

In *P. anserina*, the defects in the number and size of ascospores produced in an ascus can result from defective meiotic nucleus segregation, which leads to uneven packaging of nuclei into ascospores. In this fungus, both meiotic divisions occur along the long axis of the ascus, resulting in four equally spaced nuclei upon meiosis completion (Fig. 9A). At the following interphase (interphase-II in Fig. 9A), each pair of second-division sister nuclei migrate toward each other, producing two pairs of nuclei placed at opposite sides of the ascus (Fig. 9B). These nuclei then undergo mitosis with a division plane perpendicular to the ascus long axis, which results in four pairs of closely associated nonsister nuclei (postmeiotic mitosis in Fig. 9A). Upon ascospore delineation, each of these pairs of nuclei becomes included within a common ascospore (Fig. 9A). As a result, most asci contain four even-sized binucleate ascospores (Fig. 9C). In a small percentage of asci (≈2%), one of the four binucleated ascospores produced is replaced by two small uninucleate ascospores (Fig. S1) (55–59).

We analyzed nuclear distribution in asci issued form Δ*yop1* (Fig. 9D), Δ*yop2* (Fig. 9E), and Δ*rtn1* (Fig. 9F to J) homozygous crosses. We found that the distribution of nuclei in Δ*rtn1* ascospores was uneven. Consistent with the ascospore formation defects of Δ*rtn1*, we observed that the number of nuclei of Δ*rtn1* ascospores was abnormal, in correlation with the size of the ascospores and with their number per ascus (Fig. 9H to J). In addition, we observed defects in the distribution of nuclei following meiosis (Fig. 9F) and the postmeiotic mitosis (Fig. 9G).

### Loss of RTN1 affects spindle dynamics during meiotic development.

**FIG 10:**
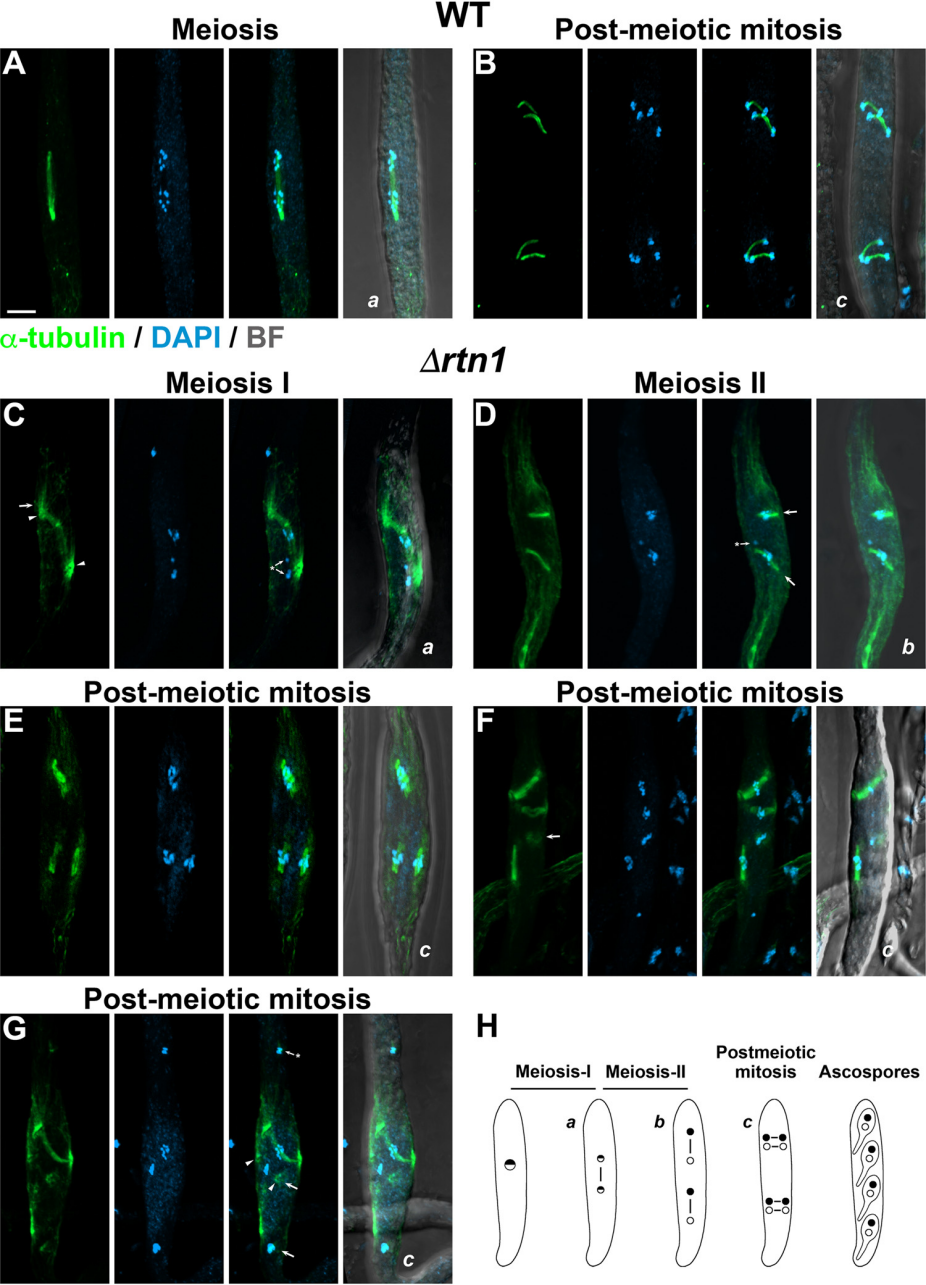
*RTN1* deletion affects spindle dynamics during meiotic development. Analysis of spindle arrangement during wild-type (A and B) and *Δrtn1* (C to G) meiotic development. (H) Schematics of spindle positioning during wild-type meiotic development. (A) Wild-type meiosis (anaphase-I) showing the spindle longitudinally oriented along the ascus. Note the 14 chromosomes (*n* = 7) migrating toward the poles. (B) WT postmeiotic mitosis (telophase) showing the spindles positioned across the ascus and aligned in pairs at opposite sides of the ascus (see Movie S6 for a 3D reconstruction). (C) *Δrtn1* first meiotic division (anaphase-I) with altered spindle positioning. Note the large array of astral microtubules (arrow; arrowheads point to the spindle poles) and the chromosomes not associated with the spindle (asterisk). (D) *Δrtn1* second meiotic division (metaphase-II) with incorrect spindle orientation (arrows); note that one spindle is perpendicular to the ascus long axis, whereas the second one is oblique. Note also a chromosome not placed in the spindle (asterisk). (E to G) *Δrtn1* postmeiotic mitosis asci. In panel E, the orientation of the four spindles is longitudinal instead of perpendicular to the ascus (note that the two upper spindles overlap; see Movie S6). In panel F, the spindles are randomly oriented and scattered along the ascus. Note also that one spindle is abnormal in shape (arrow). In panel G, one oblique spindle is observed, and a second is faintly detectable (arrows; arrowheads point to the spindle poles). The remaining two groups of chromosomes have no assembled spindles associated (small arrows). Asci in panels E to G are in metaphase. Images show maximum-intensity projections of z series through the entire cells. Small lettering indicates successive developmental stages. BF, bright field. Bar, 5 μm.

Alterations in the distribution of nuclei in ascospores in *P. anserina* can be produced by defective spindle positioning or impaired nuclear migration during meiotic development (59–61). In this fungus, the spindles are positioned along the long axis of the ascus during both meiotic divisions (shown for meiosis-I in Fig. 10A), whereas they are perpendicular to it at postmeiotic mitosis, where they are placed as two parallel pairs at opposite sides of the cell (Fig. 10B and Movie S6) (57, 58). We analyzed spindle arrangement during meiotic development and found that *RTN1* deletion produced defects in spindle orientation during both meiotic divisions (Fig. 10C and D) and postmeiotic mitosis (Fig. 10E and F and Movie S6). We also observed altered spindle positioning during postmeiotic mitosis, with spindles randomly scattered in the ascus instead of being widely separated as parallel pairs (Fig. 10F). In addition, we found defects in spindle morphology, which could reflect defective spindle assembly (Fig. 10F and G, arrows), as well as genetic material likely representing chromosomes, which were not associated with detectable spindles (Fig. 10C, D, and G, asterisks). Overall, we observed that 61.8% of *Δrtn1* asci (*n* = 34) displayed spindle arrangement abnormalities at either the meiotic (in 60% of asci, *n* = 20) or postmeiotic (in 64.3% of asci, *n* = 14) divisions, compared to 9.5% in the wild type (12% at meiosis, *n* = 25; 5.9% at postmeiotic mitosis, *n* = 17). These results show that spindle dynamics during *P. anserina* meiotic development requires RTN1.

10.1128/mBio.01615-21.10 MOVIE S6 Spindle positioning in wild-type and Δ*rtn1* postmeiotic mitoses. 3D reconstruction of the spindles in WT (first segment) and Δ*rtn1* (second segment) postmeiotic mitosis asci. In each segment, the rendered reconstruction of chromosomes (blue) is shown by moving along the *x* axis, and then the distribution of the spindles (green) is shown (WT is in telophase; Δ*rtn1* is in metaphase). Note that the spindles are positioned across the ascus in the wild type, while they are longitudinally oriented in Δ*rtn1*. The 2D projections of the 3D volumes are shown in Fig. 10B and E, respectively. Download Movie S6, MOV file, 18.1 MB. Copyright © 2021 López-Fuentes et al. 2021 López-Fuentes et al. https://creativecommons.org/licenses/by/4.0/ This is an open-access article distributed under the terms of the Creative Commons Attribution 4.0 International license.

## DISCUSSION

Here, we analyzed the function of the proteins of two main families of ER-structuring proteins—the reticulon and Yop1/DP1 families—during development of the model fungus *P. anserina*. We found that this fungus possesses three proteins of these families and found that they perform partially redundant roles during its vegetative (somatic) phase. In addition, we discovered that one of these proteins—the reticulon RTN1—is distinctively required during sexual development. This protein associates with different domains of the ER at different developmental stages, where it likely participates in different developmental processes. Our research provides relevant information on the specific mechanisms involved in ER regulation during development.

We identified three proteins of the reticulon and Yop1 families in *P. anserina* and showed that two of them—RTN1 and YOP2—prominently localize to the apical domain of the peripheral ER, while the third one—YOP1—is required for its formation. To a lesser extent, RTN1 and YOP2 were also required for the formation of this domain. This ER domain specifically associates with the polarized growing region of hyphae (47); however, our finding that the formation of this domain depends on YOP1 but that no hyphal growth defect was produced upon its elimination argues against a role for this domain in polarized hyphal growth. Nonetheless, a moderate reduction in the apical extension rate of hyphae was observed when YOP1 was eliminated in combination with RTN1 and/or YOP2, and this defect was reflected in the mycelial growth. Yet, in this case, a stronger impact was observed when the three proteins were missing, which could indicate a different arrangement of the mycelial network of the distinct mutants. These observations suggest that these proteins perform overlapping functions required for ER integrity beyond solely shaping the apical ER compartments and indicate that these functions are required for optimal vegetative growth. Still, the growth reduction observed upon elimination of these proteins was moderate, which likely reflects additional proteins involved in structuring the ER. Actually, the growth of S. cerevisiae is also only partially reduced upon simultaneously eliminating its Yop1 and reticulon (Rtn1 and Rtn2) proteins (14), and an enhanced growth defect is produced when the Lunapark family protein Lnp1 is further disrupted (62).

We also discovered that a fraction of RTN1 associates with the Spitzenkörper. The Spitzenkörper is a complex structure localized at the tip of growing hyphae, which orchestrates the polarized traffic of cell wall-building vesicles and determines hyphal growth and morphogenesis. This structure accumulates secretory vesicles before being transported to the foremost apical membrane, supplying membrane lipids and cell wall-synthesizing enzymes at the expanding hyphal tip (53, 63). As shown in Neurospora crassa, where the Spitzenkörper comprises an outer layer of glucan synthase-containing macrovesicles surrounding a core of microvesicles transporting chitin synthases, this structure accommodates distinct secretory vesicles in different strata and could regulate their differential trafficking and sorting (63–65). These vesicles are subsequently delivered to the apical plasma membrane, where the exocyst complex mediates their localized exocytosis (66). In *P. anserina* RTN1 distinctly associated with a Spitzenkörper layer likely representing the microvesicle core. It is therefore possible that RTN1 is involved in the formation and/or trafficking of some of these vesicles. In line with this hypothesis, reticulons are required for the organization of the ER exit sites, regulate protein ER exit, and contribute to ER-Golgi trafficking (26, 27, 67). Moreover, reticulons promote exocytosis by enhancing the ER-to-the cell surface trafficking (25), and they interact with exocytic SNAREs (68). Furthermore, in S. cerevisiae Rtn1 interacts with the exocyst by directly binding to its subunit Sec6 (69), which could indicate a second association of reticulons with the polar growth apparatus of fungal cells. Interestingly, proteins involved in the regulation of autophagy localize to the Spitzenkörper in Aspergillus nidulans, where they could locally modulate autophagy to regulate secretion levels (70, 71). Reticulons play different functions in autophagy, including roles in autophagosome formation (72) and ER turnover by selective autophagy (34–37). It is therefore tempting to speculate that RTN1 is involved in an autophagy-related secretory system, which could regulate secretion to modulate the cell surface membrane supply and/or cope with excess ER (73, 74). These observations suggest a role for RTN1 in the tip growth apparatus of *P. anserina*. Our finding that no growth defect was observed in *RTN1-*deleted strains could indicate redundant components involved in this role.

The RTN1 apical localization during early meiotic development suggests that it could also participate in the tip growth apparatus of asci. Notably, RTN1 redistribution to the ascus middle region following ascus growth could indicate that it performs different functions in distinct cell regions at different stages of ascus development. In the latter cell region, RTN1 could be involved in meiotic nucleus segregation, as supported by the finding that this process is compromised when RTN1 is missing. These observations also indicate that the ER undergoes a developmental remodeling during meiotic development. Interestingly, in contrast to the vegetative phase, where RTN1 seems to be partially redundant with YOP1 and YOP2, the latter proteins do not appear to be involved in meiotic development. Notably, no ascospore formation defects were observed in the plant-pathogenic fungus Fusarium graminearum upon disruption of the atlastin Sey1—a dynamin-like GTPase that mediates the fusion between the ER tubules, which generates the ER reticular network (75). These observations could indicate a specific role for RTN1 in meiotic development. Still, further research is required to elucidate the functional conservation of the ER-shaping proteins among fungi.

Our data support a role for RTN1 in the regulation of meiotic spindle dynamics. However, the precise participation of this protein in this process remains to be determined. RTN1 could be required for the organization of the NE during meiosis. In *P. anserina*, as in other ascomycetes, the NE is maintained during both meiotic divisions and postmeiotic mitosis (58). In Schizosaccharomyces pombe, meiosis involves a transient loss of the nucleocytoplasmic barrier, which occurs without NE breakdown (76–78), whereas the closed mitosis of A. nidulans involves extensive remodeling of the nuclear pore complexes, which changes their transport properties and allows timely access of tubulin and regulatory proteins to the nucleus (79, 80). Nuclear pore complexes insert into the NE at the sites where the outer and inner nuclear membranes are contiguous through highly curved membrane connections. Actually, the assembly and integrity of the nuclear pore complexes in the NE depend on the ER membrane-bending proteins (23, 81). Meiotic development in *P. anserina* could involve a remodeling of the nuclear pore complexes, requiring RTN1 and regulating the nuclear transport of proteins involved in spindle assembly and dynamics. In agreement with this hypothesis, we observed that a fraction of RTN1 localized to the nuclear periphery during meiotic development.

RTN1 might also be required for proper SPB functioning. The SPBs are microtubule-organizing centers, which are embedded in the NE and consist of two faces—facing the nucleoplasm and the cytoplasm, respectively—that serve as sites of nucleation for the spindle and the cytoplasmic microtubules, respectively (82). Nuclear distribution and spindle orientation in fungi rely on dynamic interactions and pulling forces exerted from the cell cortex on the cytoplasmic astral microtubules generated from the SPBs (83). Similar to the nuclear pore complexes, the SPBs insert into the NE at the sites where the outer and inner nuclear membranes fuse, in a process dependent on the ER membrane-bending proteins (28). In S. cerevisiae, loss of Rtn1 and Yop1 produces defects in SPB integrity, stability, and function, which result in reduced cytoplasmic microtubules and defective mitotic spindle formation. These defects lead to alterations in spindle structure, positioning, and orientation during mitosis (28). Therefore, in *P. anserina* RTN1 could be involved in promoting the assembly and stability of the SPBs during meiotic development.

In addition to microtubule assembly, the SPB plays a primordial role in ascospore formation. In S. cerevisiae, the SPB cytoplasmic face is transformed after meiosis from the site of microtubule nucleation into the vesicle docking complex (the meiosis-II outer plaque), which drives the assembly of the membrane that delineates ascospores (for review, see reference 84). In *P. anserina*, as in other Sordariales, rearrangements of the SPBs associated with ascospore formation occur following postmeiotic mitosis (57, 58, 85). Further supporting a role for RTN1 in SPB function, a number of Δ*rtn1* asci produced a single banana-shaped ascospore. Similar aberrant ascospores previously described in related fungi result from the formation of a single large ascospore wall beneath the ascus wall, which encloses all meiotic nuclear products (86). The formation of these ascospores is associated with SPB abnormalities (86) and could result from failure of the SPBs to conduct ascospore membrane formation around meiotic nuclei.

Altogether, our results show that the ER is subject to a developmental remodeling during sexual development, which involves the reticulon RTN1 and is required for proper progression of the sexual cycle. These findings add meiotic development to the number of developmental processes that rely on the activity of the ER-shaping proteins and underscore the relevance of the processes involved in shaping the ER in the regulation of cell development.

## MATERIALS AND METHODS

### Strains and culture conditions.

The strains used in this investigation are derived from the *P. anserina* “S” wild-type strain, and all analyzed strains were homokaryotic. Homologous recombination gene targeting was done in the *P. anserina* Δ*ku70* strain (87). *P. anserina* was grown on M2 minimal medium containing 1.1% dextrin as sole carbon source, and protoplasts were regenerated on RG medium. Ascospores were germinated in G medium supplemented with yeast extract (0.5%). After 36 to 48 h of growth at 27°C, mycelial explants from the center of the resulting colonies (of the relevant genotypes) were used to inoculate stock cultures (i.e., 48-h M2 colonies, radius ≈ 1 cm, stored at 4°C) and cultures for long-term stocks (i.e., explants from 48-h M2 colonies stocked in liquid RG medium at −75°C). For all experiments, to avoid alterations due to senescence or storage of the strains, cultures were inoculated with mycelial explants issued from fresh young M2 growing cultures at incubation distances <3 cm from the point of inoculation of the original ascospore yielding the corresponding strain. When required, media were supplemented with phleomycin (40 μg ml^−1^), Geneticin (G418 sulfate, 100 μg ml^−1^), nourseothricin (40 μg ml^−1^), or hygromycin B (30 or 75 μg ml^−1^, for gene tagging or deletions, respectively; see below). Current *P. anserina* methods and media can be found at http://podospora.i2bc.paris-saclay.fr.

### Nucleic acid isolation, transformation, and plasmids.

*P. anserina* genomic DNA isolation and the polyethylene glycol (PEG)-mediated protoplast transformation were performed as described in reference 88. The hygromycin resistance gene cassette used for gene deletions was derived from plasmid pBC-Hygro (89), and the Streptomyces noursei *nat1* gene from pAPI509 (a derivative from pAPI508 [87]). GFP-Hyg^R^ and mCherry-Hyg^R^ cassettes used for endogenous gene tagging were obtained from pUC-GFP and pUC-Cherry, respectively (90). pPable vector (91) was used for cloning *GFP*::*RTN1*. Plasmids GA0AA237CB11 (*P. anserina* genomic DNA library; Genoscope, France) and pSM334_Genticin were kindly provided by Robert Debuchy (Institute for Integrative Biology of the Cell, CNRS, France). Plasmid pCCG::N-GFP (92) was obtained from The Fungal Genetics Stock Center (Manhattan, KS, USA). Oligonucleotide primers used in this research are listed in Table S1 in the supplemental material.

10.1128/mBio.01615-21.4 TABLE S1 Oligonucleotide primers used in this research. Download Table S1, PDF file, 0.05 MB. Copyright © 2021 López-Fuentes et al. 2021 López-Fuentes et al. https://creativecommons.org/licenses/by/4.0/ This is an open-access article distributed under the terms of the Creative Commons Attribution 4.0 International license.

### Gene sequences.

*RTN1* (*Pa_1_22550*), *YOP1* (*Pa_2_5730*), and *YOP2* (*Pa_4_3260*) gene sequences were obtained from the *P. anserina* genome sequence (https://genome.jgi.doe.gov/Podan2/Podan2.home.html). Their predicted protein sequences are available in the GenBank database under accession numbers CDP24655.1, CDP25565.1, and CDP28238.1, respectively. FUNGIpath v.4.0 (http://fungipath.i2bc.paris-saclay.fr [93]) was used to analyze for orthologous proteins in fungal genomes.

### Gene deletions.

Mutant strains deleted for *RTN1*, *YOP1*, and *YOP2* were generated by replacing their corresponding open reading frame (ORF) with a selectable marker by homologous recombination. *RTN1* and *YOP2* were replaced by the *hph* gene, while *YOP1* was replaced by the *nat1* gene. Gene replacement cassettes were generated by double-joint PCR (94) by flanking the selectable marker gene with ≈700 bp of the 5′ and 3′ flanking sequences of the ORF to be deleted. The 5′ fragment of these constructs was amplified with primers *gene-5F* and *gene-5R* (where *gene* indicates the corresponding deleted gene [Table S1]), and the 3′ fragment with primers *gene-3F* and *gene-3R*. The respective selectable marker gene was amplified with primers *gene-hph-F* and *gene-hph-R* (*yop1-nat-F* and *yop1-nat-R*, for YOP1). The generated gel-purified fusion PCR products were used to transform *Δku70* cells, and the obtained transformants were purified after sexual crosses to the wild type. Purified homokaryotic strains of both mating types (*mat*+ and *mat*−) and on the *KU70^+^* genetic background were recovered from the progeny of these crosses. Correct gene deletions were verified by PCR analyses.

### Gene complementation analyses.

For the gene complementation assays, protoplasts of the *Δrtn1* strain were cotransformed with vector GA0AA237CB11, which harbors a copy of the *RTN1* gene, and pSM334_Genticin (in a 3:1 molar ratio). Eighteen Geneticin-resistant (Gen^R^) transformants were randomly selected and analyzed for ascospore formation. Taking into account the recessive nature of the *Δrtn1* sexual development phenotype, we inspected the restoration of ascospore formation in perithecia issued from heterozygous crosses of *Δrtn1* to each *Δrtn1*(Gen^R^) transformant (*n* ≥ 100 asci per strain). Four *Δrtn1*(Gen^R^) analyzed strains displayed a partial restoration of ascospore formation (i.e., producing between 8 and 13% of abnormal asci), and seven exhibited an ascospore formation restoration with levels of abnormal asci comparable to those of the wild type (0.8 to 3.7%). Three of these strains were further analyzed in triplicate experiments (Fig. S3).

### Tagging of RTN1 and YOP2.

We tagged RTN1 and YOP2 by fusing the coding sequence of GFP or mCherry to the 3′ end of their corresponding ORFs at their genomic loci. The tagging was attained by replacing the stop codon of *RTN1* or *YOP2* endogenous genes by a DNA cassette consisting of an in-frame fluorescent protein-encoding gene followed by a hygromycin resistance cassette (GFP-Hyg^R^ or mCherry-Hyg^R^ cassettes, respectively [90]) by homologous recombination. The tagging constructs were generated by fusion PCR and consisted of, for *RTN1*, (i) the last 720 bp (excluding the stop codon) of *RTN1* ORF 3′ end (amplified with primers *rtn1-F* and *lkt-rtn1*), fused in frame to (ii) the GFP-Hyg^R^ cassette from pUC-GFP (amplified with primers *rtn1-lkt* and *rtn1-hph-Ra*), and followed by (iii) 703 bp of DNA downstream *RTN1* stop codon (amplified with primers *hph-rtn1-Fa* and *rtn1-3R*). For *YOP2*, the construct consisted of (i) the last 619 bp of *YOP2* ORF 3′ end (excluding the stop codon amplified with *yop2-F* and *lkt-yop2*), (ii) the mCherry-Hyg^R^ cassette from pUC-Cherry (amplified with *yop2-lkt* and *yop2-hph-Ra*), and (iii) 690 bp of DNA downstream *YOP2* stop codon (amplified with *hph-yop2-Fa* and *yop2-3R*). The fusion PCR-generated tagging cassettes were gel purified and used to transform protoplasts of a Δ*ku70* strain. Hyg^R^ transformants were randomly selected, and the Hyg^R^ marker was recovered in the *KU70^+^* genetic context after crosses to the wild type. *RTN1* and *YOP2* tagging was verified by PCR analyses and by sequencing.

Double-joint PCR was used to generate a *GFP*::*RTN1* gene to express RTN1 tagged with GFP at its N terminus. A DNA PCR fragment containing the *GFP* gene preceded by the Neurospora crassa *ccg-1* promoter was amplified from plasmid pCCG::N-GFP with primers *CCG1-F* and *rtn-GFP-R* and fused in frame to the coding sequence of the *RTN1* gene followed by 703 bp of its 3′ flanking sequence (amplified with primers *GFP-rtn-F* and *rtn1-3R*). The gel-purified fusion PCR product was cloned into plasmid pGEM-T Easy (Promega, Madison, WI), to be subcloned into the EcoRI site of plasmid pPable. The generated plasmid (pFS06) was verified by sequencing and used to transform Δ*rtn1* cells. A selected transformant was crossed to the wild-type strain, and the purified *GFP*::*RTN1* ectopic transgene was recovered from the progeny of this cross in homokaryotic strains of the Δ*rtn1* and *RTN1*^+^ genetic backgrounds, respectively.

### Vegetative growth and sexual reproduction analyses.

Mycelial growth was determined by measuring the radius of mycelial colonies growing on M2 medium at 30°C every 24 h for 5 days. For each strain, three independent experiments, each with triplicates, were performed. The growth rate was defined as the slope of the corresponding growth curves. A two-way analysis of variance (ANOVA) using a Tukey *post hoc* analysis was performed to evaluate significant differences. Sexual development was analyzed as detailed recently (95); briefly, sexual crosses were performed by growing homokaryotic strains of opposite mating type on opposite sides of an M2 plate at 27°C in constant light for 3 days, after which they were cross-fertilized by spreading 2 ml of water over the colonies. Sexual cycle cells were obtained from perithecia issued from these crosses 4 days after fertilization.

### Cytology.

Sexual cycle cells were fixed in 7.4% paraformaldehyde and processed for fluorescence microscopy as described in reference 85. The ER was visualized using an ER-targeted GFP, which contained the ER targeting and retention signals of the putative *P. anserina* ER chaperone BiP (ER-GFP) (47). Nuclei and mitochondrial DNA were stained with DAPI (0.5 μg ml^−1^), and the Spitzenkörper with the styryl dye FM4-64 [*N*-(3-triethylammoniumpropyl)-4-(6-(4-(diethylamino) phenyl) hexatrienyl) pyridinium dibromide, 7 μM] (Molecular Probes, Eugene, OR). To visualize the spindle apparatus, cells were labeled with mouse monoclonal (DM1A) anti-alpha-tubulin antibody (Abcam, Cambridge, United Kingdom; ab80779; dilution 1:1,000), using either fluorescein isothiocyanate (FITC)-conjugated (Jackson ImmunoResearch, West Grove, PA; 715-095-150; dilution 1:100) or Alexa Fluor 594-conjugated (Jackson ImmunoResearch; 715-585-150; dilution 1:100) donkey anti-mouse secondary antibodies. Live-cell microscopy was performed on growing leading hyphae from entire *P. anserina* colonies grown for 24 h on agarose beds of M2 medium containing 0.55% dextrin (90). To analyze the apical extension rate of individual hyphae, growing leading hyphae were imaged at 2-s intervals for 3 min using a 63× objective in a temperature chamber at 30°C. In the resulting time-lapse series, a segmented line tracing the trajectory of the hyphal tip across time was manually drawn, and a kymograph based on this line was plotted using the Reslice command of ImageJ. The slope of the kymograph line corresponding to the extending hyphal tip was used to determine the hypha apical extension rate. For each strain, 21 hyphae issued from 3 biological replicates (7 hyphae/replicate) were analyzed. The analysis of abundance of the apical ER subcompartments was done on confocal micrographs of the midplane of growing leading hypha expressing ER-GFP. For each strain, 40 hyphae issued from 4 biological replicates (10 hyphae/replicate) were analyzed. For each hypha, GFP images were thresholded using the Maximum Entropy algorithm and the area occupied by each apical ER patch was determined using the Particles analyzer in ImageJ. Statistical significance was determined by two-way ANOVA with Tukey’s multiple-comparison test.

### Microscopy.

Epifluorescence microscopy was performed on a Nikon Eclipse E600 microscope using a cooled Neo Andor scientific complementary metal oxide semiconductor (sCMOS) camera. Confocal microscopy was done in a Zeiss LSM800 inverted laser scanning confocal microscope with a Plan-Apochromat 63×/1.4 oil immersion objective using 405-, 488-, and 561-nm laser lines. Bright-field images were acquired using the Electronically Switchable Illumination and Detection (ESID) module. For live-cell confocal microscopy, an equivalent Zeiss LSM800 system equipped with a temperature chamber (at 27°C) was used, and images from all channels were captured simultaneously. For three-dimensional (3D) imaging, z-section images were collected at 0.37- to 0.43-μm intervals through entire cell volumes. Images were processed using Zen 3.1 software (Carl Zeiss, Jena, Germany) or the FIJI package for ImageJ (96, 97).
