# Supplementary material for: Spindle Dynamics during Meiotic Development of the Fungus Podospora anserina Requires the Endoplasmic Reticulum-Shaping Protein RTN1
Source: mBio. 2021 Oct 5;12(5):e01615-21. doi: 10.1128/mBio.01615-21 (PMC8546617; doi:10.1128/mBio.01615-21)
Supplement: FIG S1 [file mbio.01615-21-sf001.pdf]

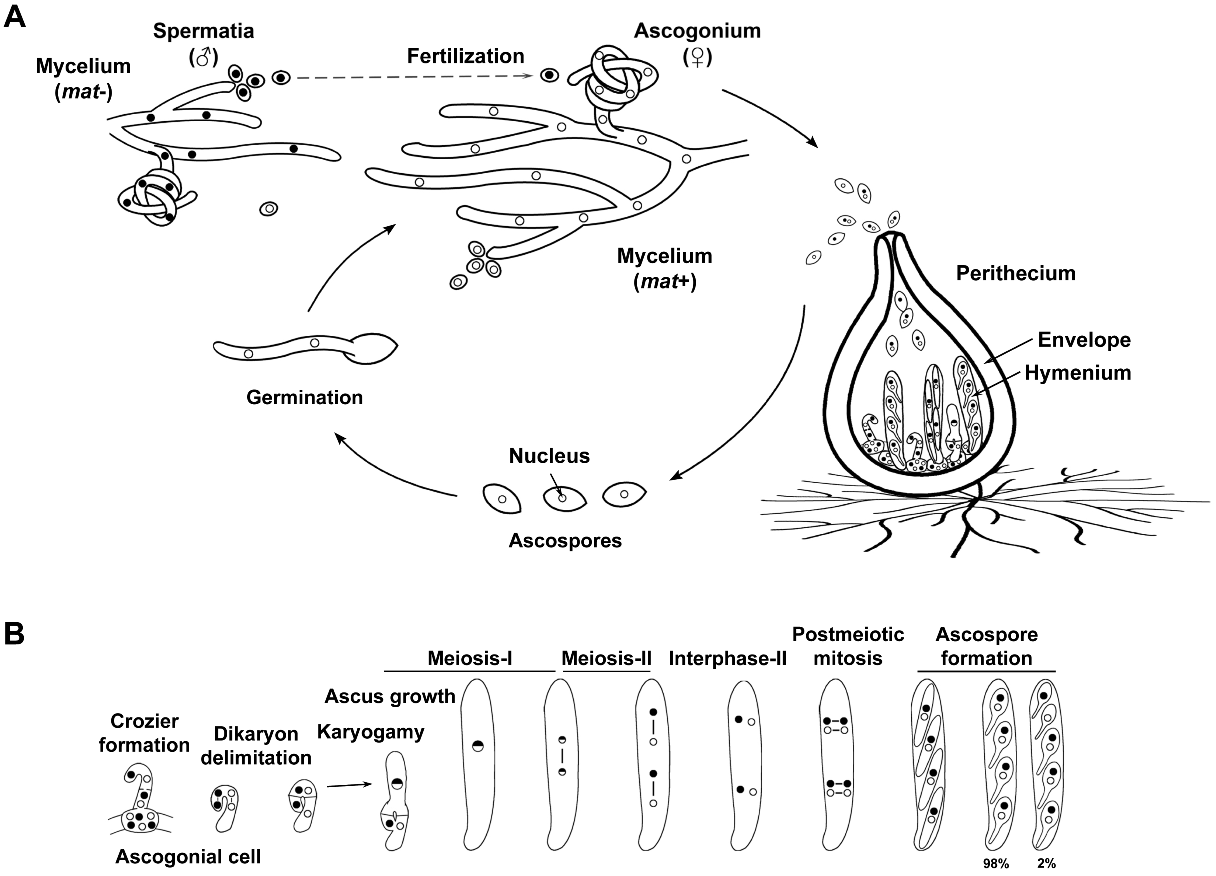

FIG S1 (A) *Podospora anserina* life cycle. *P. anserina* is an heterothallic ascomycete that reproduces exclusively sexually. Sexual reproduction takes place between genetically compatible strains differing in their mating type (denoted as *mat*+ and *mat*−, respectively, and illustrated by nuclei [dots] with different shading), and involves the formation of multicellular fructifications known as perithecia. The perithecium arises from the fertilization of a female gametangium (ascogonium) by a male gamete (spermatium) and consists of a pyriform structure that encloses the fertile tissue (the hymenium) where karyogamy, meiosis and ascospore (the meiosis-derived spore) formation occur. Ultimately, ascospores are expelled out of perithecia to germinate and produce a mycelium. Note that *P. anserina* produces binucleate (dikaryotic) and uninucleate (homokaryotic) ascospores (see below); for clarity, only the life cycle of homokaryotic strains issued from uninucleate ascospores is depicted. (B) *P. anserina* sexual development from the dikaryotic stage to ascospore formation (from left to right). The hymenium arises from the fertilized ascogonial cells, which differentiate specialized hook-shaped cells called croziers. In the crozier, the sorting and synchronized mitosis (lines connecting nuclei depict spindles) of a pair of opposite mating-type nuclei followed by the formation of septa in the site formerly occupied by the spindles produce a dikaryotic cell. This cell suffers karyogamy, enters meiosis and differentiates into an ascus (the meiocyte). Asci then elongate from about 5 to more than 150 µm along meiotic prophase-I. Following meiosis, each nucleus divides mitotically to yield eight nuclei, which are subsequently packaged by pairs into four ascospores. Since the *mat*+/*mat*− alleles (idiomorphs) very frequently (≈98%) segregate at the second meiotic division, and because of the meiotic/postmeiotic spindle positioning, most dikaryotic ascospores produced possess opposite mating-type nuclei and yield, upon germination, a heterokaryotic self-fertile mycelium. This mating behavior is known as pseudohomothallism. In a low proportion of asci (≈2%), the change in the orientation of one of the postmeiotic mitosis spindles results in two distant nonsister nuclei, which are individually packaged into independent ascospores. This results in asci containing three binucleate ascospores and two smaller uninucleate ascospores. Ascospore differentiation and maturation are completed inside the original ascus.
