# Supplementary material for: Spindle Dynamics during Meiotic Development of the Fungus Podospora anserina Requires the Endoplasmic Reticulum-Shaping Protein RTN1
Source: mBio. 2021 Oct 5;12(5):e01615-21. doi: 10.1128/mBio.01615-21 (PMC8546617; doi:10.1128/mBio.01615-21)
Supplement: FIG S2 [file mbio.01615-21-sf002.pdf]

A

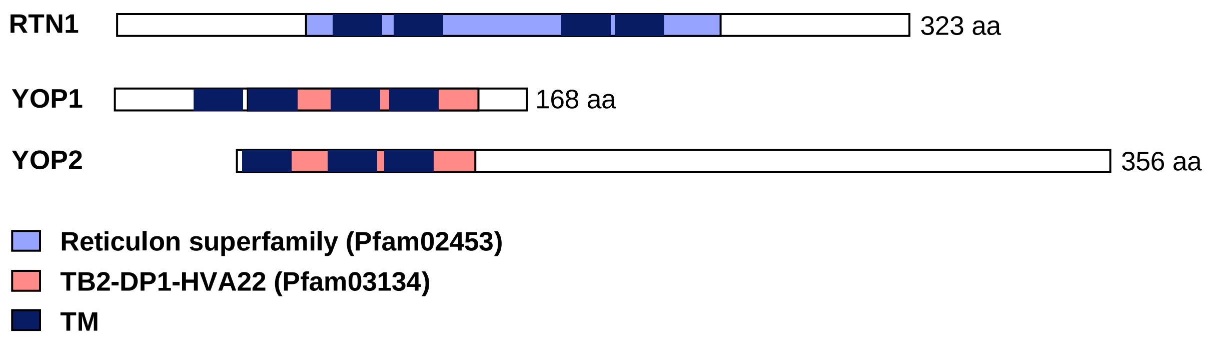

B

– RTN1–

```
P.anserina   MADISN-GHANGNASVDALKNNLAATYNNVTSGPVAQNIKAEGARTTDELSNLANSRRAPSYTAATGQPL 69
A.nidulans   MADNSEVRYPDPNANIK----------ESITNGPVAETIRSEASRTGQEFRDLKSAKVIPSTTTATGQPL 60
S.cerevisiae ---------------------------------------------------------MSASAQHSQAQQ- 13
H.sapiens    ----------------------------------------------------------------MDGQK- 5
A.thaliana   ------------------------------------------------------------MANDVTKDP- 9

                                      TM1                           TM2
P.anserina   THYHSFFSELLSWKNPRASGIAYLTIVSFIFSVRYLDVLRWGLKLTWMALGVTIAAEIAGKAILNNGFAT 139
A.nidulans   TYYHSLFYSLLSWEQPRATAASFASVVAFIFAARYLPLLRWFFKFLYLVIGVTAAAEIGGRVVLSQGLTS 130
S.cerevisiae QQQKSCNCDLLLWRNPVQTGKYFGGSLLALLILKKVNLITFFLKVAYTILFTTGSIEFVSKLFLGQGLIT 83
H.sapiens    KNWKDKVVDLLYWRDIKKTGVVFGASLFLLLSLTVFSIVSVTAYIALALLSVTISFRIYKGVIQAIQKSD 75
A.thaliana   TPKSDIVEDIYLWRRKKLAFSTLLVSTSTWILLSFYGFTTITI-VSWIGIAVVSMIFLWGSLL---RLLS 75

                             RHD                                     TM3
P.anserina   QLRPRKYYT-----VPRETLDAVIGDVNELINFGVIESQRILF---AENIWASA-AIALGAFISYYLVKV 200
A.nidulans   SFRPRKYYT-----LPRETIEAVLEDFQQLVDFLFIEFQRILF---AENAVHTT-AAAAAAFSAYWLIKI 191
S.cerevisiae KYGPKECPN-----IA-GFIKPHIDEALKQLPVFQAHIRKTVF---AQVPKHTF-KTAVALFLLHKFFSW 143
H.sapiens    EGHPFRAYLESEVAISEELVQKYSNSALGHVNCTIKELRRLFL---VDDLVDSL-KFAVLMWVFTYVGAL 141
A.thaliana   KVEPELSGL--------EVSEEFVVETVRSCRMLMEEMVRWMFRVGAESEWFVFARTVLGFWILSRIGNL 137

                     TM4
P.anserina   VPYWGLALIATSVIEFAPLIYTTNQELIDSQIQHAGEIINDQTEQIRSLVQKNTEQATQVTKQYMGDYTA 270
A.nidulans   LPFWGLSLLAVTIAYLGPFVYLNNQEIIDAQIENVRQMVGSQANQLKDLAEERTYHATGVVKQYVGDYSN 261
S.cerevisiae FSIWTIVFVADIFTFTLPVIYHSYKHEIDATVAQGVEISKQKTQEFSQMACEKTKPYLDKVESKLGPISN 213
H.sapiens    FNGLTLLILALISLFSVPVIYERHQAQID---------------HYLGLANKNVKDAMAKIQAKIPGLKR 196
A.thaliana   LDFHTCLFIGLVMGLTVPKLWEEYGDQIQ---------------KHLGSLKDKSKGAYNTTHEKILEMKN 192

P.anserina   KAQSLI---------------KGAVGQENGHKSELKPTDFPVAPKEDIKSEPIAPPTKAG---------- 315
A.nidulans   KAQEYIGRRSASPEVAKGPAAGPVVKREPEPEAVVKTSDFPEAPKVEPVAQSIETQS------------- 318
S.cerevisiae LVKSKTAPVSSTAGPQTASTSKLAADVPLEPESKAYTSSAQVMPEVPQHEPSTTQEFNVDELSNELKKST 283
H.sapiens    KAE------------------------------------------------------------------- 199
A.thaliana   KLH------------------------HGTEEKVKKSE-------------------------------- 206

P.anserina   -----EEEPLIAA 323
A.nidulans   -----EKEPLLAI 326
S.cerevisiae KNLQNELEKNNAX 296
H.sapiens    ------------- 199
A.thaliana   ------------- 206
```

C

– YOP1–

```
                                                                          TM1            TM2
Pa YOP1       MSSPQDKAQVYVGQLDKELSKYPVLINLEKQTGVPKAYAVLGSVALYFFLILLNLG--GQLLTNIAGFVI 68
An AN2279     MASFQDRAQHTIAQLDKELSKYPVLNNLERQTSVPKVYVILGLGGIYTFLVFFNIA--GQLLVNLAGFIL 68
Sc Yop1p      MSEYASSIHSQMKQFDTKYSGNRILQQLENKTNLPKSYLVAGLGFAYLLLIFINVGGVGEILSNFAGFVL 70
Yl Yop1       MSQIIDQVQAALQNIDKELEKYPALKELEKQIPVPKSYILLGFVGFYFILIFLNIGGIGQLLSNIAGLVI 70
Hs DP1        MRERFDRF------LHEKNCMTDLLAKLEAKTGVNRSFIALGVIGLVALYLVFGYG--ASLLCNLIGFGY 62
At HVA22D     ------------------------------------------MDKFWTFLTALHSG-----AGPIVMLLY 23

Pa YOP2       -----------------------------------------------MFDIFAKL------LSSIASFLF 17
An AN6059     ----------------------------------------------MFFGIFADL------LSSILTILF 18
YlYALI0E05841 --------------------------------------------------MFHALG-------SIASVVF 13
Hs REEP1      -----------------------------------------------MVSWIISRL-----VVLIFGTLY 18
AtHVA22-like  -----------------------------------------------MIGSFLTRG-----LVMVLGYAY 18

                    TB2_DP1_HVA22
                                         TM3                     TM4
Pa YOP1       PGYYSLGALFS--ADKHDDTQWLTYWVVFSFFTVLESFISV-VYWVPFYYTFKFIFLLWLSLPS-FKGAE 134
An AN2279     PTYYSLDALFS--AGKADDTQWLTYWVVYAFFTVVESAISA-PYWFPFYYIFKFALVLWLALPQ-TNGAQ 134
Sc Yop1p      PAYLSLVALKT--PTSTDDTQLLTYWIVFSFLSVIEFWSKAILYLIPFYWFLKTVFLIYIALPQ-TGGAR 137
Yl Yop1       PGYYSLLALET--PGKADDTQYLTYWVVFATLNVFEFWSKAILYWVPFYYLFKTAFLLYIGLPQ-YGGAE 137
Hs DP1        PAYISIKAIES--PNKEDDTQWLTYWVVYGVFSIAEFFSDIFLSWFPFYYMLKCGFLLWCMAPSPSNGAE 130
At HVA22D     PLYASVIAMES--TTKVDDEQWLAYWIIYSFLSLTELILQSLIEWIPIWYTVKLVFVAWLVLPQ-FQGAA 90

Pa YOP2       PLFASYKALKT--SDPAQLTPWLMYWVVLACALLVESWTEWFLCWIPFYAYLRFFFLLYLVLPQ-TQGAR 84
An AN6059     PIFASYKALRS--SDPYQLAPWLMYWVVLSAILMAESWTYFIIGWIPFYSWIRLGFFAYLVLPQ-TQGAR 85
YlYALI0E05841 PIFASYRAIKS--HDMTYATPWLKYWVVMGIEQALENTFGVVLSVLPLYSLARLVFFAWLVLPQ-SQGAV 80
Hs REEP1      PAYYSYKAVKS--KDIKEYVKWMMYWIIFALFTTAETFTDIFLCWFPFYYELKIAFVAWLLSPY-TKGSS 85
AtHVA22-like  PAYECYKTVEKNRPEIEQLRFWCQYWILVACLTVFERVGDAFVSWVPMYSEAKLAFFIYLWYPK-TRGTT 87

Pa YOP1       IIFRSFLAPTLSRH-------------------------------------------------------- 148
An AN2279     IVFKSLVQPLVGRY-------------------------------------------------------- 148
Sc Yop1p      MIYQKIVAPLTDRY-------------------------------------------------------- 151
Yl Yop1       LVYKAIVKPLAQKL-------------------------------------------------------- 151
Hs DP1        LLYKRIIRPFFLKHESQMDSVVKDLKDKSKETA------------------------------------- 163
At HVA22D     FIYNRVVREQFKKH-------------------------------------------------------- 104

Pa YOP2       YIYEEYVHPRLEENETAIEELIASAHDRLKAAGVAYLKQAIEYLKTNILGFPPSPDAASSASASQPTQPQ 154
An AN6059     ILYQDYVEPFLAHHEREIEEFIGRAHERAKALGLQYLYQAIDFVRERVLGLPPQRPPTPP--------PA 147
YlYALI0E05841 RLYDEKVEPFLDRYNTQIEDFFANGHTYVRDYGLQYLSVLVKWLTGKGFDTEAAKSTSANPAPATPVVPQ 150
Hs REEP1      LLYRKFVHPTLSSKEKEIDDCLVQAKDRSYDALVHFGKR------------------------------- 124
AtHVA22-like  YVYESFFRPYLSQHENDIDHSLLELRTRAGDMAVIYWQRVASYGQTRILEILQYVAAQSTPRPQPPQKRG 157

Pa YOP1       ---------------------------------------------------------------------- 148
An AN2279     ---------------------------------------------------------------------- 148
Sc Yop1p      ---------------------------------------------------------------------- 151
Yl Yop1       ---------------------------------------------------------------------- 151
Hs DP1        ---------------------------------------------------------------------- 163
At HVA22D     ---------------------------------------------------------------------- 104
Pa YOP2       TPQSYTQSLLAKFTLPSARWGSTAGATPPSHTASLGSDFYSFLASAVSAAASASAAPSTP-TPQQQRPDS 223
An AN6059     SAASYAQSLLSRFNLPSA-----VGGTNPAPA----NDWYSAISSAVAAVTSPGKS----HESRADELSA 204
YlYALI0E05841 SYMDSVMGYIKSNSPAT-------------------GGKITRLFDMYRAAGAVGGAVGSREVTETVEIPS 201
Hs REEP1      ---------------------------------------------------------------------- 124
AtHVA22-like  GRANQAPAKPKKAPVPQ-------------------S------------------------EPEEVSLSS 184
```

C **– YOP1 (cont.)–**

```
Pa YOP1        --------------------------------------------------FQTSGST------------- 155
An AN2279      ---------------------------------------------------FTGGST------------- 154
Sc Yop1p       ------------------------------------------------ILRDVSKTE------------- 160
Yl Yop1        -----------------------------------------------VNIQPHGGP-------------- 160
Hs DP1         -------------------------DAIT----------------------------------------- 167
At HVA22D      ----------------------------------------------GVLRSTHSKPT------------- 115
Pa YOP2        WSSIIPPSVRGAGASARMSFIQAQRERLNIVMSALDREEQAALHDNRLGSVSQDGSPGSASGLSKSRSEQ 293
An AN6059      SGSLLPREFESKTRAEKASFYSKQRDMLDVLRNALINEERNLEHAEEDPLAYGGGAP-----LRKNRSDN 269
YlYALI0E05841  SLPHSEQLGLIDKERSRLLAALSSLDARGSLLKSDTKSKSNASLASGVNITASDYSPLLASSSDTEFDVV 271
Hs REEP1       --------GLNVAATAAVMAASKGQGALSERLRSFSMQDLTTIRGDGAPAPSGPPPP------------- 173
AtHVA22-like   SSSSSSSENEGNEPTRKVSGPSRPRPTVTSVPAADPKNAGTTQIAQKSVASPIVNPP----QSTTQVEPM 250

Pa YOP1        -------------------------------------ASGLRAKADLHTE-------------------- 168
An AN2279      -------------------------------------SANLRAQADAATKSQ------------------ 169
Sc Yop1p       -------------------------------------KDEIRASVNEASKATGASVH------------- 180
Yl Yop1        -------------------------------------SDSLKAQAQSAVDAAESHVPQGHSTGVSH---- 189
Hs DP1         -------------------------------------KEAKKATVNLLGEEKKST--------------- 185
At HVA22D      -------------------------------------KPNILHSIFPHREGHEAHSH------------- 135
Pa YOP2        DFEKLEAESGEEDEPRVHRRQVPERGTSTGGWMPWAWGQGGNTAGAGGKKEEQGRSSGFDGGQ------- 356
An AN6059      SFDHIEHEDTRDRSPR-----------RSGNW----FGDGEQTGAASGVEFAMRTVDAIARAREAR---- 320
YlYALI0E05841  KSEDVLGLDGNVKDERPNSRGWFWQRHYSKVGEAN--KAGYEKLVDPPSEPPVSGASMARESEEEEPLIP 339
Hs REEP1       -------------------------------------GSGRASGKHGQPKMSRSASESASSSGTA----- 201
AtHVA22-like   QIEEVEGEAESGNE------------------NPN--PEGPKETVMEETIRMTRGRLRKTRSEESR---- 296

Pa YOP1        --- 168
An AN2279      --- 169
Sc Yop1p       --- 180
Yl Yop1        --- 189
Hs DP1         --- 185
At HVA22D      --- 135
Pa YOP2        --- 356
An AN6059      --- 320
YlYALI0E05841  SNL 342
Hs REEP1       --- 201
AtHVA22-like   --- 296
```

FIG S2 Sequences of the *P. anserina* proteins of the reticulon and Yop1 families. (A) Schematics of *P. anserina* RTN1, YOP1, and YOP2. Note that RTN1 and YOP1 possess characteristic RHD (Pfam02453) and TB2/DP1, HVA22 family (Pfam03134) domains, respectively, characterized by two large hydrophobic segments, each predictably composed of two tandem transmembrane domains. In contrast, YOP2 possesses an N-terminal TB2/DP1, HVA22 domain, in which only one transmembrane domain could be detected in the first of its two hydrophobic segments. (B) Alignment of RTN1 with representative proteins from fungi, humans, and plants. The reticulon homology domain (RHD) and the putative transmembrane-spanning regions (TM) of P. *anserina* RTN1 are overlined (in light and dark blue, respectively). The transmembrane segments of *Saccharomyces cerevisiae* (Y. Shibata, C. Voss, J. M. Rist, J. Hu, et al., J Biol Chem 283:18892-

18904, 2008, https://doi.org/10.1074/jbc.M800986200), *Homo sapiens* (G. K. Voeltz, W. A. Prinz, Y. Shibata, J. M. Rist, and T. A. Rapoport, Cell 124:573-586, 2006, https://doi.org/10.1016/j.cell.2005.11.047; N. Zurek, L. Sparks, and G. Voeltz, Traffic 12:28-41, 2011, https://doi.org/10.1111/j.1600-0854.2010.01134.x), and *Arabidopsis thaliana* (E. Breeze, N. Dzimitrowicz, V. Kriechbaumer, R. Brooks, et al., Proc Natl Acad Sci U S A 113:10902-10907, 2016, https://doi.org/10.1073/pnas.1605434113; I. Sparkes, N. Tolley, I. Aller, J. Svozil, et al., Plant Cell 22:1333-1343, 2010, https://doi.org/10.1105/tpc.110.074385) proteins are underlined in green. GenBank accession numbers of the protein sequences utilized are as follows: *Podospora anserina*, CDP24655.1; *Aspergillus nidulans*, QDK59803.1; *S. cerevisiae*, Rtn1p, AAS56905.1; *H. sapiens* reticulon-4 isoform C, NP_008939.1; *A. thaliana* Reticulon-like protein B13, NP_565555.1. (C) Alignment of *P. anserina* YOP1 family proteins with representative proteins from fungi, humans, and plants. The TB2_DP1_HVA22 family domain (red) and the putative transmembrane-spanning regions (dark blue) of *P. anserina* YOP1 and YOP2 are overlined. The transmembrane segments of *S. cerevisiae* Yop1p (J. P. Brady, J. K. Claridge, P. G. Smith, and J. R. Schnell. Proc Natl Acad Sci U S A 112:E639-648, 2015, https://doi.org/10.1073/pnas.1415882112) are underlined (green). GenBank accession numbers of the protein sequences utilized are as follows: *P. anserina* (Pa) YOP1, CDP25565.1; YOP2, CDP28238.1; *A. nidulans* (An) AN2279.2 (Yop1), XP_659883.1; AN6059.4 (Yop2), CBF70269.1; *S. cerevisiae* (Sc) Yop1p, NP_015353.1; *Yarrowia lipolytica* (Yl) YALI0_B19668g (Yop1), XP_501105.2; YALI0_E05841g (Yop2), XP_503603.1; *H. sapiens* (Hs) DP1, AAA60136.1; REEP1 isoform 2, NP_075063.1; *A. thaliana* (At) HVA22 homologue D, NP_567713.1; HVA22-like protein I, NP_568606.1. Sequence alignments were performed using MUSCLE (R. C. Edgar. Nucleic Acids Res 32:1792-1797, 2004, https://doi.org/10.1093/nar/gkh340). Identical amino acids are shaded in black (shade threshold 60%). The proteins were analyzed for conserved domains on the Pfam database (pfam.xfam.org), and the putative transmembrane-spanning regions were predicted using TOPCONS (K. D. Tsirigos, C. Peters, N. Shu, L. Kall, and A. Elofsson. Nucleic Acids Res 43:W401-407, 2015, https://doi.org/10.1093/nar/gkv485), with similar result using Philius (S. M. Reynolds, L. Kall, M. E. Riffle, J. A. Bilmes, and W. S. Noble. PLoS Comput Biol 4:e1000213, 2008, https://doi.org/10.1371/journal.pcbi.1000213) (not shown).
