# Supplementary material for: Spindle Dynamics during Meiotic Development of the Fungus Podospora anserina Requires the Endoplasmic Reticulum-Shaping Protein RTN1
Source: mBio. 2021 Oct 5;12(5):e01615-21. doi: 10.1128/mBio.01615-21 (PMC8546617; doi:10.1128/mBio.01615-21)
Supplement: FIG S3 [file mbio.01615-21-sf003.pdf]

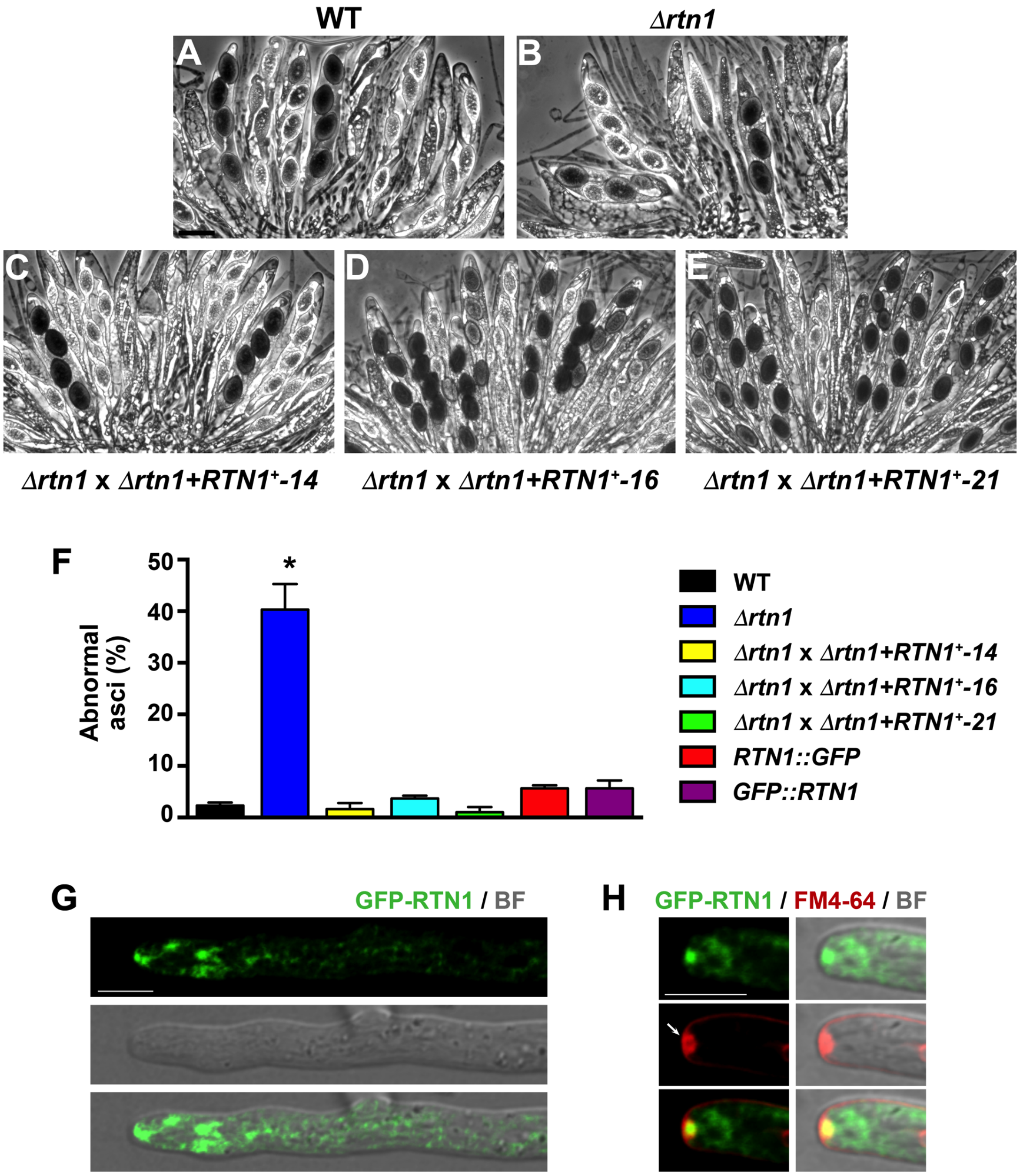

FIG S3 Genetic complementation of *Δrtn1* strains and analysis of the strains expressing RTN1 tagged with GFP. Analysis of ascospore formation in homozygous sexual crosses of WT (A) and Δ*rtn1* (B) strains and in heterozygous crosses of Δ*rtn1* to Δ*rtn1* strains complemented with a wild-type *RTN1*$^{+}$ allele (Δ*rtn1+RTN1*$^{+}$; three independent transformants are shown) (C-E). Bar, 20 μm. (F) Quantitation of the abnormal asci produced in WT, Δ*rtn1* and *RTN1::GFP* homozygous crosses, and in heterozygous crosses of Δ*rtn1* to Δ*rtn1* strains complemented with a wild-type *RTN1*$^{+}$ allele (Δ*rtn1+RTN1*$^{+}$; three independent transformants were analyzed) or with a *GFP::RTN1*$^{+}$ allele (n = 300 from three independent experiments). *$P < 0.0001$ by one-way ANOVA with Tukey's *post hoc* test. (G) Localization of GFP-RTN1 expressed in an *RTN1*$^{+}$ growing leading hypha. (H) Compared apical localizations of GFP-RTN1 and the FM4-64-stained Spitzenkörper. Arrow points to the Spitzenkörper. BF, bright field. Bar, 5μm.
