# Supplementary material for: Spindle Dynamics during Meiotic Development of the Fungus Podospora anserina Requires the Endoplasmic Reticulum-Shaping Protein RTN1
Source: mBio. 2021 Oct 5;12(5):e01615-21. doi: 10.1128/mBio.01615-21 (PMC8546617; doi:10.1128/mBio.01615-21)
Supplement: TABLE S1 [file mbio.01615-21-st001.pdf]

Table S1. Oligonucleotide primers used in this research.

Primer name	Primer sequence 5’>3’
rtn1-5F	GTGACCGCAGGTAGCTGAAGAAAGA
rtn1-5R	ATGATTCTTCTCGCTTCCGGGCGGGGATTATTGGTA
rtn1-3F	CCTTCTTTCTAGCTAGAGGAGCAAGGCTTCCTTCGAC
rtn1-3R	GCTGTCTGAGCTCGTCCTTTCGTTA
rtn1-hph-F	TACCAATAATCCCCGCCCGGAAGCGAGAAGAATCAT
rtn1-hph-R	GTCGAAGGAAGCCTTGCTCCTCTAGCTAGAAAGAAGG
yop1-5F	CCACGAAACCAGAGAATTGGATGTC
yop1-5R	GAAGCATTTATCAGGGTGATGCTGCTAGCCGGTGT
yop1-3F	GTATTCAACATTTCCGTGTCGGTTGCTGGACCTCTC
yop1-3R	TGTAAGTGCAGATAGACAACTGGAG
yop1-nat-F	ACACCGGCTAGCAGCATCACCCTGATAAATGCTTC
yop1-nat-R	GAGAGGTCCAGCAACCGACACGGAAATGTTGAATAC
yop2-5F	ACGATGGAGATGGATATGTGGTTGG
yop2-5R	ATGATTCTTCTCGCTTCCTGATCCGGTATGTTGGCG
yop2-3F	CCTTCTTTCTAGCTAGAGGCAGCAGGATCAACAACGAGA
yop2-3R	CTTCCCCTCCCCAAACAAAAGAAAT
yop2-hph-F	CGCCAACATACCGGATCAGGAAGCGAGAAGAATCAT
yop2-hph-R	TCTCGTTGTTGATCCTGCTGCCTCTAGCTAGAAAGAAGG
rtn1-F	TCTCAAGCTCACATGGATGGCCCTC
lkt-rtn1	TAAACCAGCACCGTCACCAGCAGCGATCAAAGGCTC
rtn1-lkt-F	GAGCCTTTGATCGCTGCTGGTGACGGTGCTGGTTTA
rtn1-hph-Ra	GAAGGAAGCCTTGCTCACTTATTCCTTTGCCCTCGGA
hph-rtn1-Fa	TCCGAGGGCAAAGGAATAAGTGAGCAAGGCTTCCTTC
yop2-F	TACCCAACCTCAAACTCCGCAGTCA
lkt-yop2-R	TAAACCAGCACCGTCACCTTGTCCCCCATCAAACCC

yop2-lkt-F	GGGTTTGATGGGGGACAAGGTGACGGTGCTGGTTTA
yop2-hph-Ra	GTTAAAAAGACCATCTCACATCTATTCCTTTGCCCTCGGA
hph-yop2-Fa	TCCGAGGGCAAAGGAATAGATGTGAGATGGTCTTTTTAAC
CCG1-F	ATCGAATTCGTTCAAAGCCACATCA
rtn-GFP-R	TCCGTTCGAAATGTCGGCGTTAATTAATGGCGCGCC
GFP-rtn-F	GGCGCGCCATTAATTAACGCCGACATTTCGAACGGA
